# Evolutionary, structural and expression analysis of core genes involved in starch synthesis

**DOI:** 10.1038/s41598-018-30411-y

**Published:** 2018-08-24

**Authors:** Jianzhou Qu, Shutu Xu, Zhengquan Zhang, Guangzhou Chen, Yuyue Zhong, Linsan Liu, Renhe Zhang, Jiquan Xue, Dongwei Guo

**Affiliations:** 1 The key Laboratory of Biology and Genetics Improvement of Maize in Arid Area of Northwest Region, Ministry of Agriculture, College of Agronomy, Northwest A&F University, Yangling, 712100 Shaanxi China; 2 Maize Engineering Technology Research Centre of Shaanxi Province, Yangling, 712100 Shaanxi China

## Abstract

Starch is the main storage carbohydrate in plants and an important natural resource for food, feed and industrial raw materials. However, the details regarding the pathway for starch biosynthesis and the diversity of biosynthetic enzymes involved in this process are poorly understood. This study uses a comprehensive phylogenetic analysis of 74 sequenced plant genomes to revisit the evolutionary history of the genes encoding ADP-glucose pyrophosphorylase (AGPase), starch synthase (SS), starch branching enzyme (SBE) and starch de-branching enzyme (DBE). Additionally, the protein structures and expression patterns of these four core genes in starch biosynthesis were studied to determine their functional differences. The results showed that AGPase, SS, SBE and DBE have undergone complicated evolutionary processes in plants and that gene/genome duplications are responsible for the observed differences in isoform numbers. A structure analysis of these proteins suggested that the deletion/mutation of amino acids in some active sites resulted in not only structural variation but also sub-functionalization or neo-functionalization. Expression profiling indicated that AGPase-, SS-, SBE- and DBE-encoding genes exhibit spatio-temporally divergent expression patterns related to the composition of functional complexes in starch biosynthesis. This study provides a comprehensive atlas of the starch biosynthetic pathway, and these data should support future studies aimed at increasing understanding of starch biosynthesis and the functional evolutionary divergence of AGPase, SS, SBE, and DBE in plants.

## Introduction

Starch is the predominant reserve form of carbohydrate and energy in plants and can be divided into two types, transitory starch and storage starch, based on biological function. In photosynthetic tissues, transient starches accumulate in chloroplasts during the day. During the night, they are then transported and degraded to provide energy and nutritional substances for growth and metabolism. In non-photosynthetic tissues, such as seed endosperm, tubers and storage roots, storage starches are kept for long periods of time in specialized plastids termed amyloplasts, from which they can be remobilized in preparation for germination, sprouting or regrowth^1^. Additionally, starch is an important resource for agriculture, human consumption and industry. For example, it is a major contributor to the harvestable starch-storing organs of crop plants, which include cereal seeds (e.g., maize), tubers (e.g., *Solanum*) and storage roots (e.g., *Ipomoea*). Moreover, starch provides abundant calories for human diet and animal feed and is an economical, biodegradable and renewable industrial raw material^2^.

Starch consists of two types of polysaccharides: amylose and amylopectin. Amylose is a linear polymer composed of α-1,4-linked glucan chains and has very few branches connected by α-1,6-glycosidic bonds. The amylopectin molecule is larger than the amylose molecule and contains abundant α-1,6-branches that connect α-1,4-linked glucan chains and make up a structural framework of repeated amorphous and crystalline lamellae^3^. Linear amylose adjacent parallel side chains are distributed in the semi-crystalline matrix formed by amylopectin, and this organization underlies the semi-crystalline structure of starch^1^. Thus, the amylose:amylopectin ratio has a major influence on the appearance and structure of starch granules and also affects the quality of crop storage organs, food production and industrial applications.

**Figure 1:**
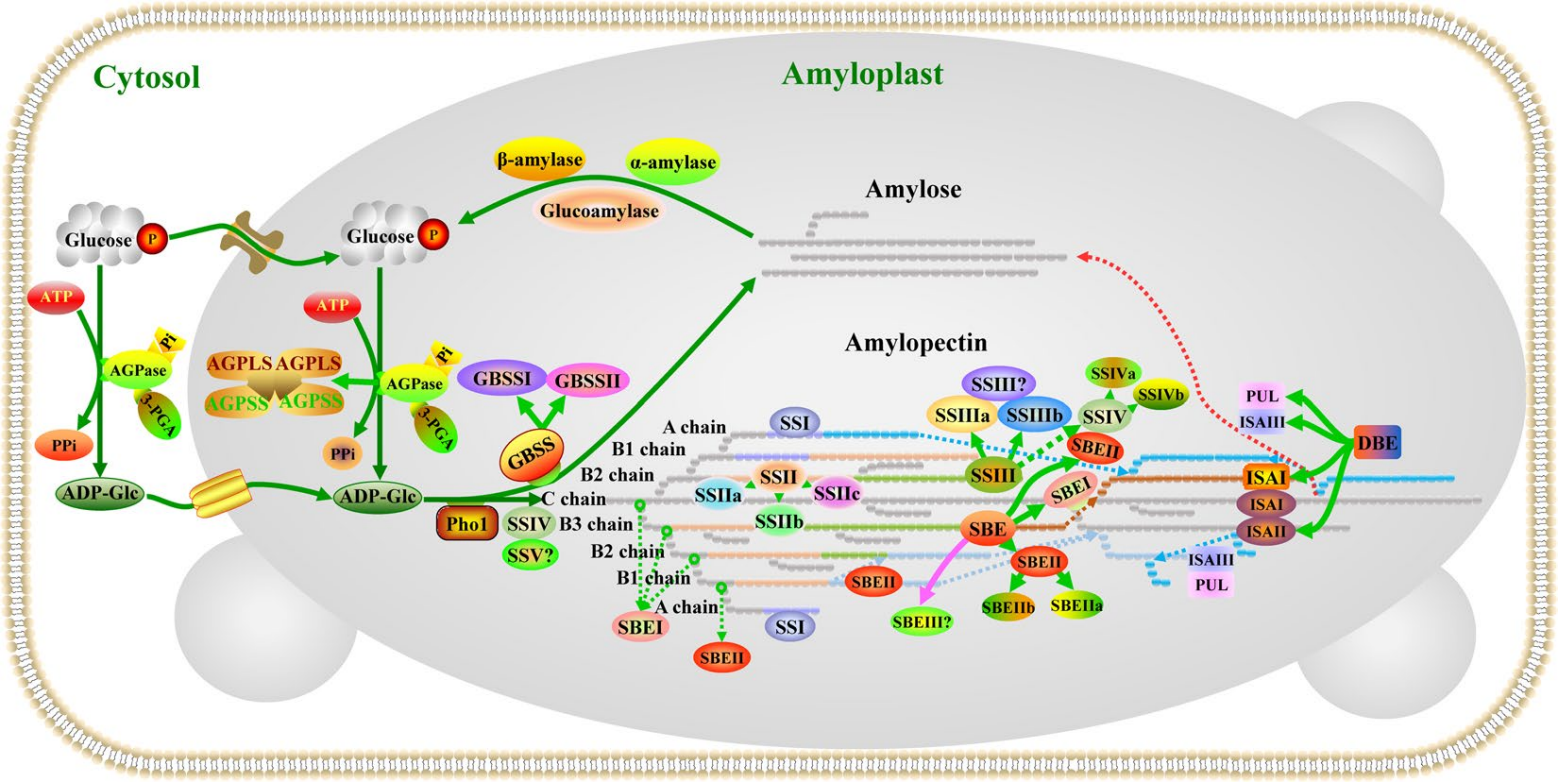
The starch biosynthesis pathway. AGPase synthesizes ADP-glucose from Glc1P and ATP and as a heterotetramer (L_2_S_2_) consisting of two large and two small subunits; AGPSS plays a catalytic function, while AGPLS is mainly responsible for modulating the allosteric regulatory properties of AGPase^4,68^. Amylose is mainly produced via the activity of GBSS. Amylopectin synthesis depends on coordinated interactions among at least 17 different genes encoding isoforms of SS, SBE, ISA, PUL and PHO1. Of these, SSI plays an important role in elongating short chains from a degree of polymerization (DP) of 6–7 chains at the branch point to DP 8–12 in the A or B1-chains of amylopectin^56,69^. SSII plays a distinct role in catalysing the formation of intermediate chains (usually DP 13–25) of amylopectin^70^. SSIII mainly catalyses the synthesis of amylopectin B2 to B4 chains, and some of its functions overlap with those of SSII in amylopectin biosynthesis^71,72^. SSIV plays an essential role in the priming of starch granule formation, the morphology of starch granules and the degree of starch accumulation; moreover, its functions can be partially supported by SSIII depending on the plant species^57,58^. SBEI preferentially produces longer chains (B1 to B3), while SBEIIa and SBEIIb preferentially promote the production of short amylopectin chains (DP 6–12) and further impact the structure and phenotype of amylopectin during starch biosynthesis^73,74^. The ISAI homomultimer and/or ISAI/ISAII heteromultimer have a higher affinity for relatively long external branches and a greater impact on amylopectin structure, while ISAII may be indirectly involved in de-branching because it recognizes special branch points and facilitates the ability of ISAI to remove nearby branches^15,34,75^. Additionally, ISAIII partially compensates for the function of the ISAI/ISAII heteromultimer and plays a major role in starch breakdown by de-branching short external chains of glucans as well as influencing the activity of α-amylase and β-amylase^76,77^. PUL has partially overlapping functions with ISA and is involved in cleaving short branched chains during starch biosynthesis^60,78^. Here, different colours represent different enzymes involved in starch synthesis. The dotted line represents the shift in the direction of the chain. The question mark indicates that the specific function of the enzyme in starch synthesis is unknown.

Starch biosynthesis is a complex and highly regulated process that requires coordinated activities among multiple enzymes, including ADP-glucose pyrophosphorylase (AGPase), starch synthase (SS), starch branching enzyme (SBE) and starch de-branching enzyme (DBE) (Fig. 1). AGPase, as the first enzyme in the starch biosynthesis pathway, catalyses the limiting reaction by converting glucose 1-phosphate (Glc-1-P) and ATP to ADP-Glc and inorganic pyrophosphate (PPi) in amyloplasts. The enzyme’s catalytic activity is stimulated by 3-phosphoglyceric acid (3-PGA) and inhibited by inorganic phosphate (Pi). The activity of AGPase is also limited by the oxidation-mediated formation of disulfide bridges between adjacent AGPSSs, which can lead to re-activation by reduced thioredoxin (or dithiothreitol *in vitro*)^4,5^. SS can be further divided into granule-bound starch synthase (GBSS), which is responsible for the synthesis of amylose and the extra-long-chain fraction of amylopectin, and soluble starch synthase (SSS), which is mainly responsible for the synthesis of amylopectin^6,7^. SBEs belong to the α-amylase family, the branching activity of which is regulated by Q-enzyme, which introduces a branched structure by cleaving the α-1,4-glucan chain in polyglucans and then reattaching the cleaved chain onto an acceptor chain via an a-1,6-glucan linkage, thereby creating a branch in the same or another chain^8^. DBEs are another glucan-modifying enzyme that occurs in two forms, namely, isoamylase-type DBE (ISA) and pullulanase-type DBE (PUL). The most important functional difference between these forms is that ISA generally acts upon phytoglycogen and amylopectin by hydrolysing the α-1,6-linkages of polyglucans, which play important roles in the modification of excessively branched chains or the removal of improper branches of amylopectin formed by branching enzymes to maintain the cluster structure of amylopectin. Moreover, ISA likely provides branched chains for amylose. PUL usually cleaves the α-1,6-linkages of polyglucans in pullulan and, to a lesser degree, amylopectin, and exerts little or no activity towards glycogen^9^. Recent studies have suggested that the plastidial pathway of starch synthesis exists in all extant higher plants and green algae and that the starch biosynthetic enzymes of higher plants underwent a complex sequence of changes during evolution^10^. Moreover, the isoform types and functionality of starch biosynthetic enzymes are remarkably similar to those found in green algae^10^. This similarity indicates that in starch biosynthetic enzyme genes, the functional regions or sites that control starch synthesis are relatively well-conserved because these lineages diverged from a common ancestor.

In plants, AGPase subunits share a common nucleoside triphosphate (NTP) transferase domain, which allows AGPase to transfer nucleotides from one compound to another, providing substrates for starch biosynthesis. SSs share a highly conserved core region located in the C-terminus that generally consists of conserved starch catalytic glucosyl transferase family 5 (GT5) and GT1 domains, which mediate an inverting mode of glucosyl transfer during glucosyl transferase^11^. Both the GT5 and GT1 domains belong to the GT-B superfamily according to the CAZy database (http://www.cazy.org/), and they possess conserved amino acid residues that can bind the glucosyl donor (ADP-Glc). These enzymes usually merge into the base catalytic region of starch synthases. All SBEs and DBEs belong to glycoside hydrolase family 13 (GH13), an important member of clan GH-H, which is also known as the α-amylase family (Aamy). These enzymes not only share an Aamy domain but also possess conserved carbohydrate-binding module family 48 domains in the N-terminal sequence. Moreover, SBEs have retained a C-terminal β-sheet catalytic domain (Aamy_C), while pullulanase (PUL) has a DUF3372 domain located in the N-terminal sequence, and this domain may play important roles in recognition and/or interaction with certain substrates or be involved in modulating PUL activity and interacting with other starch biosynthetic enzymes in specific environmental conditions^12,13^.

Many previous studies have reported enzymes that are directly involved in starch biosynthesis in algae^14^, potato^15^, *Arabidopsis thaliana*^16^, barley^17^, wheat^18,19^, and rice^20,21^. However, far less research has been devoted to the core regulatory network involved in starch metabolism. This paucity of research means that limited information is available to assist breeders and biotechnologists in improving and increasing starch content in a predictable manner. In the present study, we present and discuss the starch synthesis network, which is regulated by multiple starch biosynthetic enzymes, and evolutionary patterns in starch biosynthetic enzymes in 74 plant genomes. By combining data related to the structures, functions and expression patterns of maize starch biosynthetic enzyme genes, we reveal previously undetected information about starch biosynthetic enzymes and the starch synthesis network. It is anticipated that these results will enhance our understanding of the starch synthesis process.

## Results

### Phylogenetic, structural and functional sites analysis of AGPase subunits

**Figure 2:**
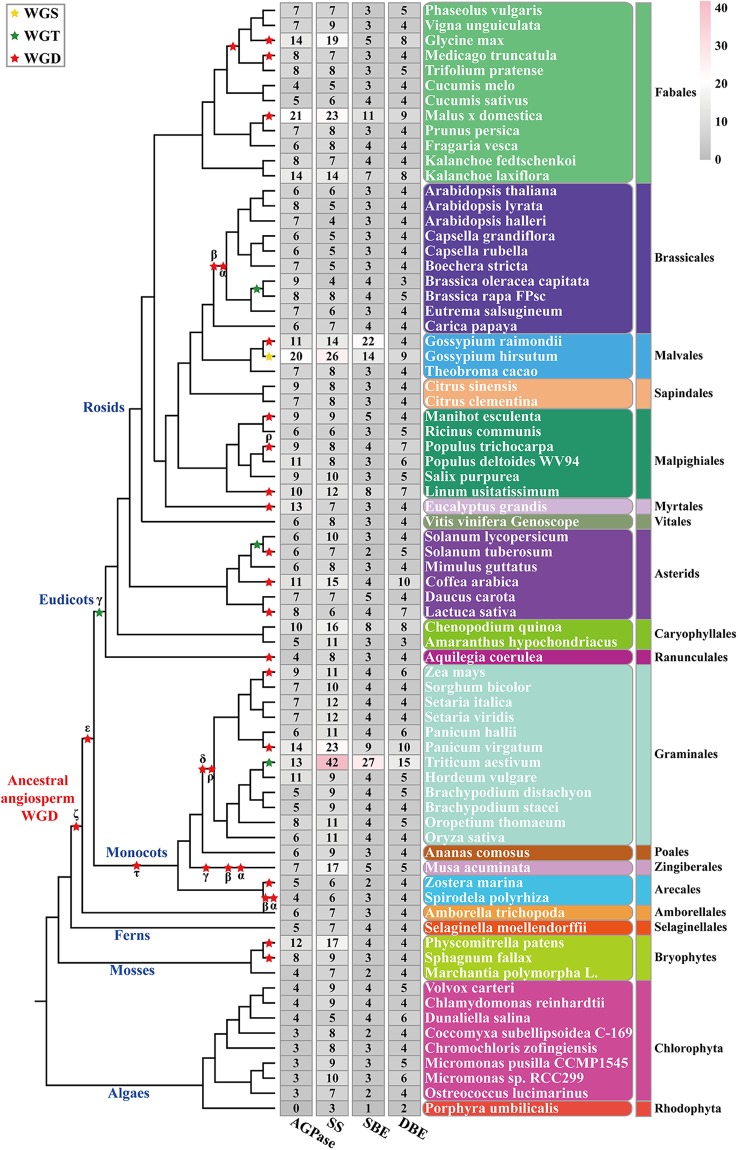
Branch-specific expansion of AGPase, SS, SBE and DBE genes in every sub-group on every branch of the phylogenetic tree. The tree on the left displays all polyploidy events (marked with stars). Red stars represent whole-genome duplication events, green stars represent whole-genome triplication events, and yellow stars represent whole-genome sextuplication events. The total number of protein isoforms of the four core enzymes and the number in each group identified in each plant genome are indicated on the right. Species names are shown on the right side.

**Figure 3:**
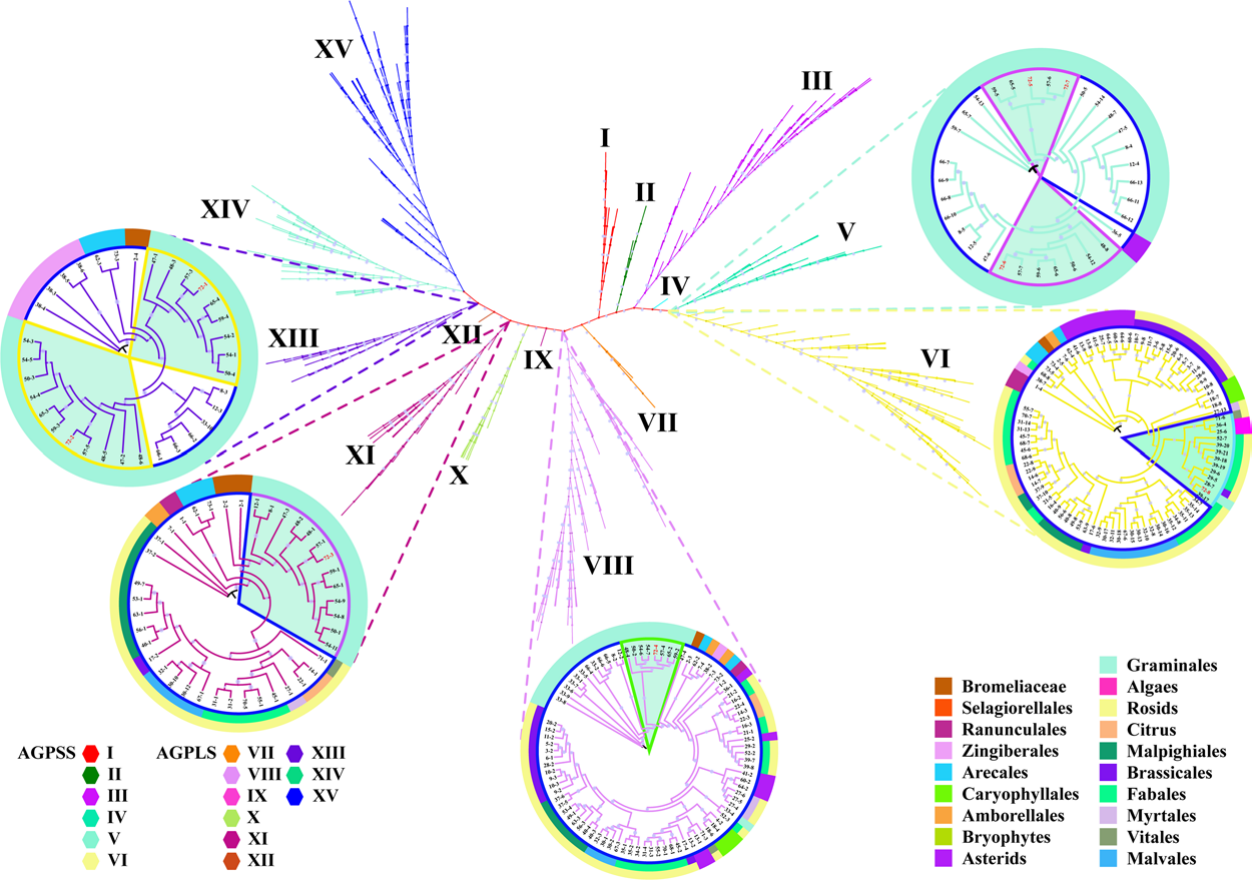
Phylogenetic analysis of the amino acid sequences of AGPase of 74 plant species. Sequences classified into subfamilies I-XV are shown in colour. Of these, I-VI and VII-XV represent the AGPSSs and AGPLSs, respectively, found in the 73 plant species. The branches of protein sequences in maize are highlighted in red with a light blue background. As an example, we have illustrated the internal divergence between AGPLS and AGPSS. For detailed species ID and protein sequences, please refer to Supplementary Table 1.

All AGPase-encoding genes have been identified in many plant genomes (Fig. 2). The phylogenetic tree of AGPase subunits from 73 plant species showed that there is considerable evolutionary divergence in these plant species (Supplementary Table 1). In particular, we did not detect similar AGPase-encoding genes in the *Porphyra umbilicalis* genome (Fig. 2). This result echoed previous studies^2,22,23^. Additionally, the evolution of AGPLSs was markedly different from that of AGPSSs in the studied plant species, and this difference may have arisen as a result of different duplication events and selection pressures between AGPLSs and APGSSs^24,25^ (Fig. 3 and Supplementary Fig. 1).

**Figure 4:**
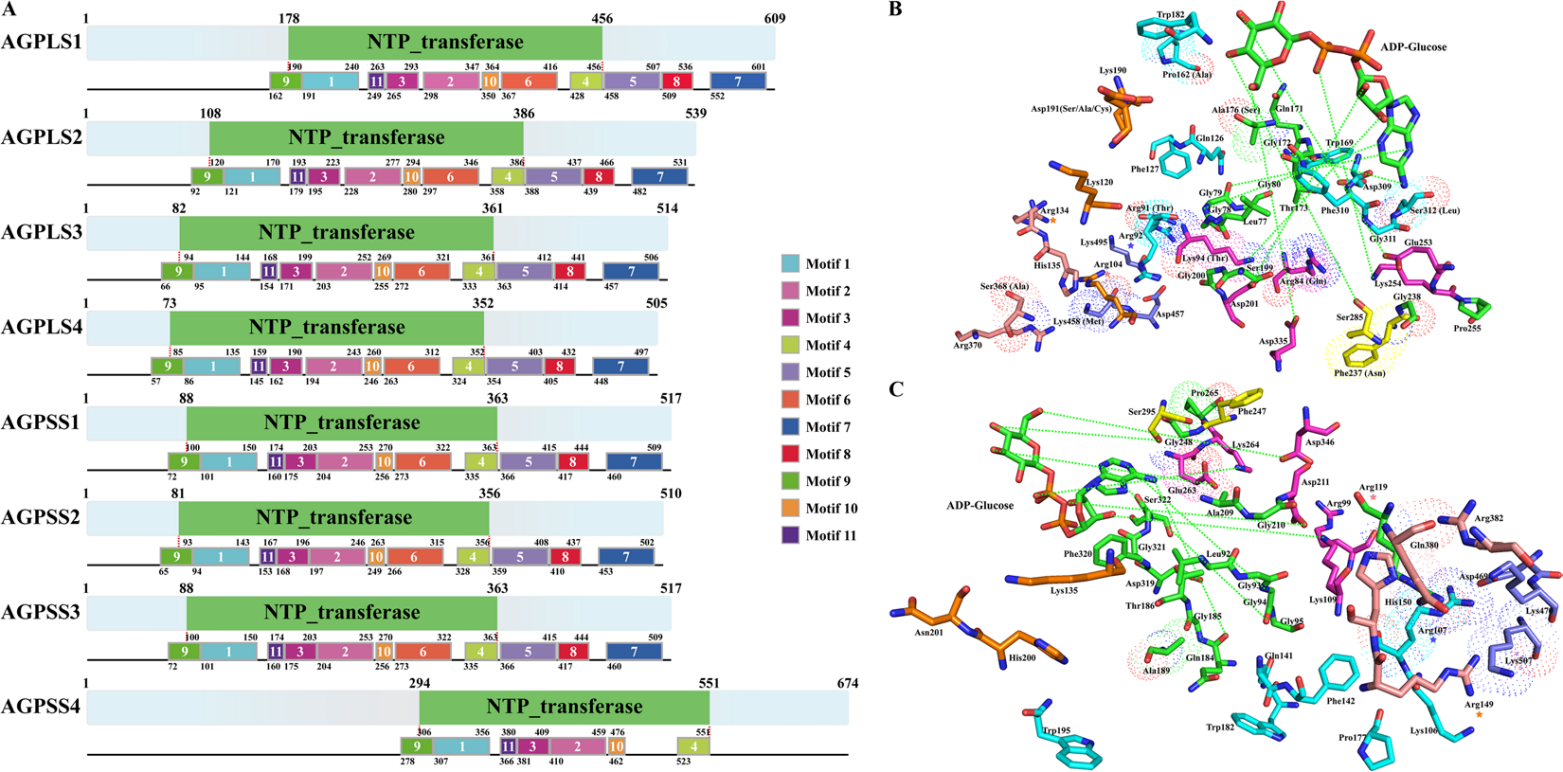
Structure and active site analysis of AGPase subunits. Maize is shown as an example. (**A**) The composition and distribution of domain structures and conserved motifs in AGPase proteins are marked and annotated in different colours. Based on the conservation of functional sites, we selected representative subunits for analysis. (**B**) Stereo view of the active sites of AGPLSs based on the sequence of AGPLS4 and (**C**) of AGPSSs based on the sequence of AGPSS1. Interaction sites between AGPSSs/AGPLSs and ADP-glucose are linked by broken green lines. Different colour stars and amino acids represent different functional sites. The same site with different amino acids is marked with dots in AGPase subunits.

Although there has been a divergence between AGPLSs and AGPSSs during evolution, they share a core region of approximately 31 kDa that is indispensable for catalytic activity and is often designated the NTP_transferase (nucleotidyl transferase) domain (Fig. 4A). An analysis of conserved motifs revealed that motif 6 was not detected in the NTP_transferase domain of AGPSS4, while other AGPase subunits all contained motifs 1, 2, 3, 4, 6, 9, 10 and 11 (Fig. 4A and Supplementary Fig. 2). Additionally, a secondary structure analysis showed that there were more α helices and β sheets in the catalytic domain of AGPSSs than in the catalytic domain of AGPLSs (except for AGPSS4, Fig. 4A and Supplementary Fig. 3).

To further explore the active sites for AGPase subunits, we aligned the protein sequences of maize AGPase subunits with those of potato sequences (sequence similarity was greater than 46%)^26^. Sulfate is an inhibitor of the potato tuber ADP-Glc PPase α subunit homotetramer under certain conditions^26^. Three areas were found that could interact with the sulfate in maize AGPase subunits. The first sulfate-binding area involved Arg107, Arg119, Asp469, Lys470 and Lys507; the Lys470 was replaced by Met in AGPLS1 and AGPLS2 and by His313 in AGPSS4 (Fig. 4B,C and Supplementary Fig. 4). In the second area, five residues participated in sulfate binding, including Arg149, His150, Gln380, and Arg382 and a double-active site at Arg119; Gln380 was substituted by Ala and Ser in AGPLS1 and AGPLS2-4, respectively (Fig. 4B,C and Supplementary Fig. 4). In the third sulfate-binding area, Lys135, Arg149, His200 and Asn201 participated in binding sulfate, His200 was substituted with a Lys in AGPLSs, while Asn201 was a highly variable site that was replaced by Ser, Ala, Cys or Asp in four AGPLSs (Fig. 4B,C and Supplementary Fig. 4). Additionally, multiple ATP-binding sites in maize AGPSSs were consistent with those found in potato except for AGPSS4, and Arg84, Pro162, Ala176, Ser199 and Ser312 had changed in AGPLSs (Fig. 4B and Supplementary Fig. 4). Glucose, a major substrate for starch synthase, binds with multiple residues of AGPase that are conserved among AGPase subunits, except for Asn248 of AGPLS1 and Tyr 250 of AGPLS2 (Fig. 4B and Supplementary Fig. 4). Compared to AGPSSs, the ADP-Glc binding site Lys94 was replaced by Thr in AGPLS1 and AGPLS2 (Fig. 4B and Supplementary Fig. 4). In agreement with a previous study on potato AGPase, we found that multiple amino acid residues on the active sites of maize AGPase subunits were consistent with those found in potato AGPSS and that the interactive residues of AGPSSs were more conserved than were those of AGPLSs^26^ (Supplementary Fig. 4).

### Phylogenetic, structural and functional divergence of starch synthesis genes

**Figure 5:**
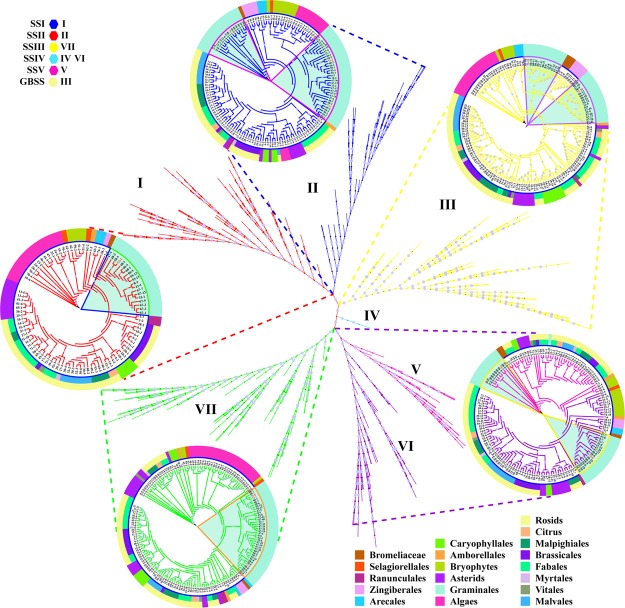
Phylogenetic analysis of the amino acid sequences of SS in 74 plant species. Sequences classified into subfamilies I-VI are shown in colour. Clades I, II, III, IV and VI/V/VII represent SSI, SSII, GBSS, SSIV and SSV, respectively, of 74 plant species. The branches of protein sequences found in maize are highlighted in red with a light blue background. For detailed species IDs, please refer to Supplementary Table 1.

To explore the evolutionary relationships of SSs, a phylogenetic tree was constructed using SS-encoding protein sequences collected from 74 plant species (Fig. 2). The phylogeny was classified into six different clades, of which clades I, II, III, IV, V and VI typically represent SSII, SSI, SSIV, SSIII, SSV and GBSS, respectively, according to the maize SS isoforms (Supplementary Fig. 5). The phylogenetic analysis showed that in most species, SS isoforms have undergone gene duplication to different degrees, and SSV demonstrated a close evolutionary relationship with SSIV (Supplementary Fig. 5 and Fig. 5).

**Figure 6:**
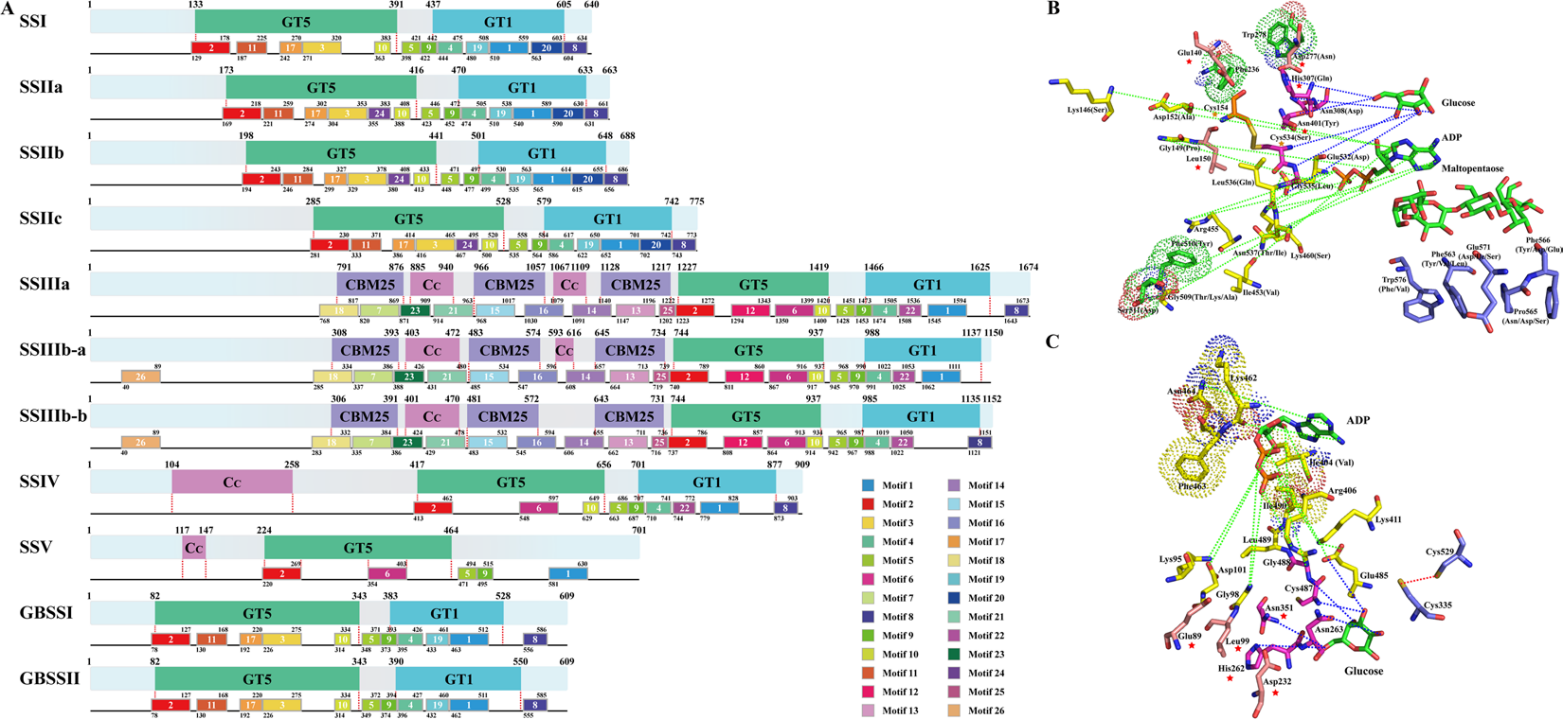
Structural features and active site analysis of SS isoforms. Maize is shown as an example. (**A**) Compositions and distributions of domain structures and conserved motifs of SS proteins are marked and annotated in different colours. (**B**) Stereo view of the active sites of SS isoforms based on the sequence of SSI and (**C**) GBSS isoforms based on the sequence of GBSSI. The same site with different amino acids is marked with dots in SS isoforms. Interaction sites between SSs and ADP are shown as linked broken green lines. Interaction sites between SSSs and glucose are marked in pink. Red stars and lines shown in light pink represent catalytic sites. Amino acid sites that interact with maltopentaose are marked in blue, and these active sites are not conserved in SSIII, SSIV and SSV. Additionally, disulfide bonds were found only in SSI and GBSSI and are marked with orange stars in SSSs and as blue amino acids in GBSSI.

The domain analysis of SS isoforms found that GT5 and GT1 domains were detected in almost all SS isoforms except SSV. The SSIII, SSIV and SSV isoforms contained one or two coiled-coil domains in the N terminus that are involved in regulating protein-protein interactions^27^ (Fig. 6A). Additionally, three conserved carbohydrate-binding modules (CBM53 domain) were detected in the N-terminal regions of SSIII isoforms that play important roles in substrate binding^28^ (Fig. 6A).

The secondary structures of maize SS isoforms were constructed based on the reference models of wheat SSI (83% identity with maize SSI) and rice GBSSI (62–83% identity, A2Y8X2.2)^17,29^. Comparative studies revealed that the main divergence in secondary structure between the SSI and SSII isoforms was in the GT5 domain, both of which were quite different from that of the SSIII isoforms with regard for the positions and compositions of α helices and β sheets (Fig. 6A and Supplementary Fig. 6). The main divergence between SSIV and SSV occurred in the GT1 domain, in which one α helix was absent in SSIV. The differences between GBSSI and GBSSII were that in the GT5 domain, one β sheet was absent in GBSSI and one α helix was absent in GBSSII, and in the GT1 domain, one β sheet was absent in GBSSII (Fig. 6A). Furthermore, an analysis of conserved motifs revealed that motif 24 was only detected in SSII isoforms, and the motif compositions were similar between SSS and GBSS isoforms except for motifs 24 and 20 (Fig. 6A and Supplementary Fig. 7). Although special motif compositions were found among SSIII isoforms (e.g., motif 1 and motif 26), they were different from the motifs found in other SS isoforms in the GT1 and GT5 domains (Fig. 6A). The distribution of motifs in the GT5 domain was similar between SSIV and SSV, but there was a significant divergence between the motifs found in the GT1 domain of these two isoforms.

To further explore the more subtle differences among SS isoforms, we aligned the maize SSS protein sequences with the reported *Escherichia coli* glycogen synthase (EcGS) and barley SSI protein sequences (Supplementary Fig. 8). The results indicated that multiple active sites were conserved in most of these sites and that the amino acid residues at these sites were involved in the combination of glucose, ADP and maltopentaose (Fig. 6B,C and Supplementary Fig. 8). In particular, the active sites of SSV were less conserved than those of other SS isoforms, and portions of the active sites were conserved and found to be similar to those of SSIII and SSIV (Fig. 6B and Supplementary Fig. 8). Moreover, variations in the amino acid residues located in active sites also caused the associative and catalytic activities of SSV to be different from those of other SSS isoforms, especially during interactions with glucose. This result indicated that SSV may not be directly involved in the extension of glucan but may instead coordinate with other SSSs to regulate the extension of glucan. Additionally, disulfide bonds were detected only in active sites in SSI and not in other SSS isoforms (Fig. 6B). These results suggest that gene duplications likely resulted in SSS isoforms accumulating higher numbers of mutations, which resulted in their sub-functionalization or neo-functionalization. Furthermore, when we compared maize GBSS isoforms with rice GBSSI, *Agrobacterium tumefaciens* glycogen synthase (AtGS) and EcGS, we found that the binding sites for ADP and glucose were conserved in maize GBSS isoforms except for residues Lys462, Phe463, Asn464 and Ile490 (Fig. 6C and Supplementary Fig. 9)^29–31^. Furthermore, the only identified inter-domain disulfide bridge was found in GBSSI, and this bridge was not present in GBSSII because the amino acid residue Ile was replaced by Val (Fig. 6C and Supplementary Fig. 9). This result suggested that a nonsynonymous substitution of amino acid residues between GBSSI and GBSSII might have caused them to diverge in spatial structure and function.

### Expansion of starch branch proteins in evolution, structure and active sites

**Figure 7:**
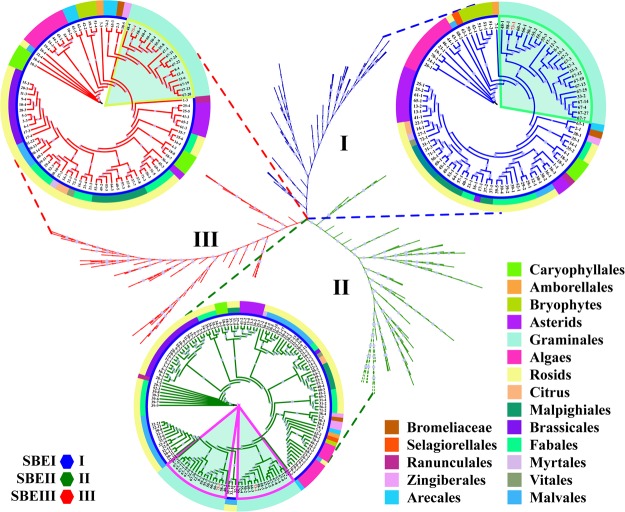
Phylogenetic analysis of the amino acid sequences of SBE in 74 plant species. Sequences classified into subfamilies I-III are shown in colour. Clade I, clade II and clade III represent SBEI, SBEII and SBEIII, respectively, in 74 plant species. The branches of protein sequences in maize are highlighted in red with a light blue background. For detailed species IDs, please refer to Supplementary Table 1.

A phylogenetic analysis of the protein sequences of SBE in 74 plant species indicated that SBE sequences could be clustered into three clades, clade I, clade II and clade III, which were designated SBEI, SBEII and SBEIII, respectively, according to the annotation for maize (Supplementary Fig. 10). In particular, almost all plant species that possessed multiple SBE isoforms were in clade II rather than clade I and clade III (Figs 2 and 7). This result indicated that the genes retained in the duplicated SBE gene pairs likely accumulated more beneficial variations than did the genes lost during evolutionary history, and this probably contributed to the diversified functions observed in the retained genes in starch metabolism (Supplementary Fig. 10 and Fig. 7).

**Figure 8:**
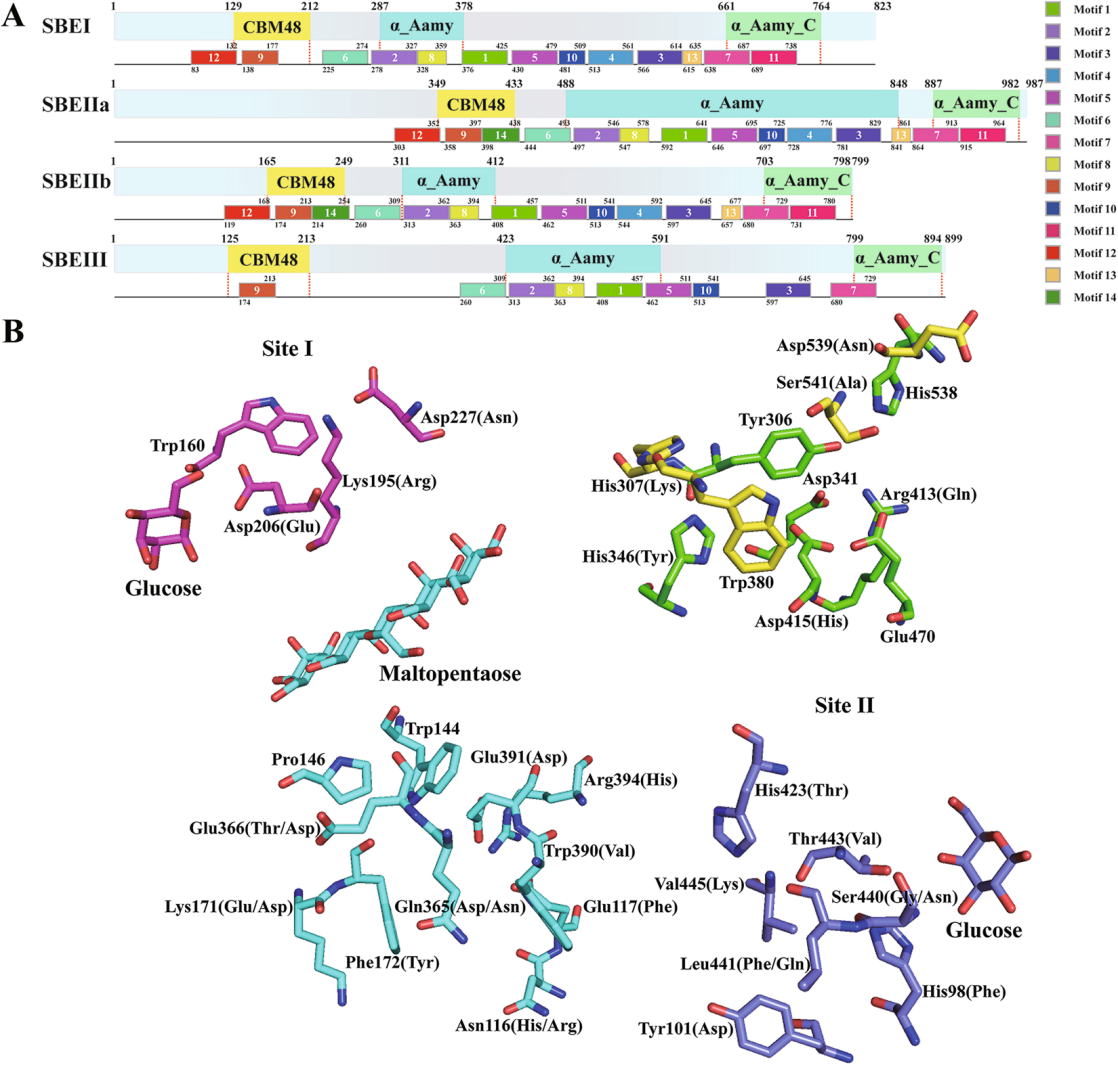
Structural and functional site analysis of maize SBE proteins. Maize is shown as an example. (**A**) Compositions and distributions of domain structures and conserved motifs of SBE proteins are marked and annotated in different colours. (**B**) Interaction site I between SBEs and glucose is shown as a magenta line. Interaction site II between SBEs and glucose are shown as light blue line, while interaction sites between SBEs and maltopentaose are shown as blue lines. The catalytic site of SBEs is shown as a green line. A yellow line indicates other active sites. Additionally, the amino acids shown in brackets represent variable sites in other SBE isoforms.

To further explore the differential features of SBE isoforms, we analysed the domain structures of the maize SBE isoforms. The domains of these SBEs were characterized by a modular architecture composed of an N-terminal domain containing a carbohydrate-binding module family 48 (CBM48) domain, a central catalytic domain (α-amylase) characterized by a (β/α)_8_-barrel, as well as an α-amylase C-terminal domain (Fig. 8A). Moreover, an overall structure model analysis demonstrated that while the secondary structures of SBEI, SBEIIa and SBEIIb were highly similar, some α helix and β sheets were absent in the CBM48 and α-amylase domains of SBEIII (Supplementary Fig. 11). Additionally, a conserved motif analysis showed that motif 14 was only present in SBEIIa and SBEIIb, and multiple motifs were not found in SBEIII (Fig. 8A and Supplementary Fig. 12).

The differences in functional regions or sites among the SBE isoforms were investigated by aligning maize SBE protein sequences with those reported for rice BEI (approximately 84% identity)^32,33^. The results indicated that some of the binding sites for maltopentaose and glucose were not conserved among SBE isoforms, and the alternative residues were mainly found in SBEIII (Fig. 8B and Supplementary Fig. 13). The catalytic sites of the maize SBE isoforms were relatively more conserved that those of rice BEI and branching enzyme (EcGBE) of *Escherichia coli*, except for Y495, Q559 and H561 in SBEIII (Fig. 8B and Supplementary Fig. 13). These phenomena indicate that SBEIII may perform different biological functions during starch metabolism than other SBE isoforms. Additionally, a comparative analysis of cyclodextrin (CD)-binding sites between EcGBE and maize SBE isoforms revealed that CD-binding sites I and V in SBEI, CD-binding sites VI and VII in SBEIIa and SBEIIb, and CD-binding sites III and IV in SBEIII were relatively conserved and that CD-binding site II was more conserved in SBEIIa, SBEIIb and SBEIII than in SBEI (Fig. 8B and Supplementary Fig. 13).

### Evolutionary, structural and functional features of starch de-branching proteins

**Figure 9:**
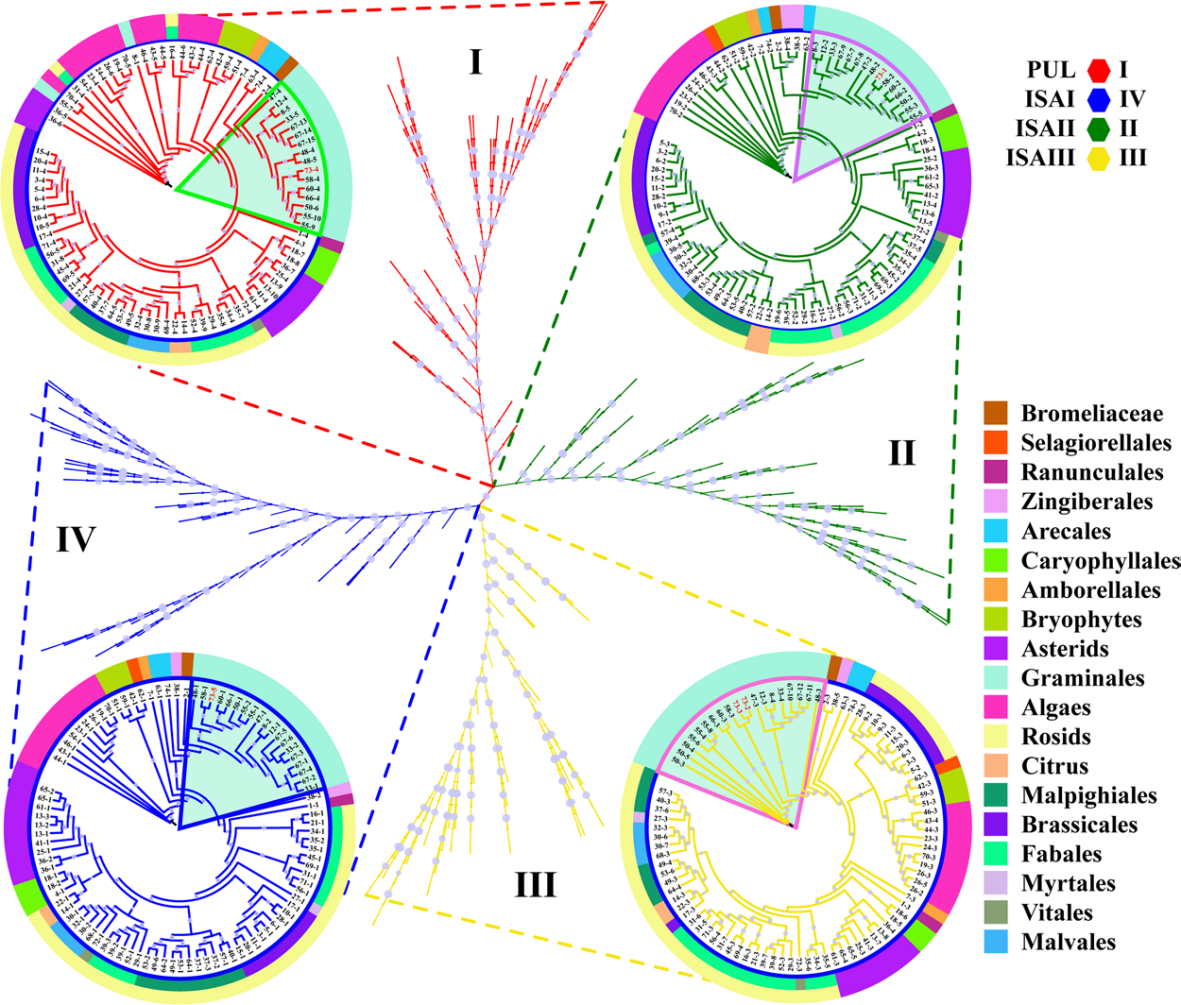
Phylogenetic analysis of the amino acid sequences of DBE in 74 plant species. Sequences classified into subfamilies I-IV are shown in colour. Clade I, clade II, clade III and clade IV represent PUL, ISAII, ISAIII and ISAI, respectively, in 74 plant species. The branches of protein sequences in maize are highlighted in red with a light blue background. For detailed species IDs, please refer to Supplementary Table 1.

DBE protein sequences from ancestral lineages of algae, mosses, ferns, monocots and eudicots were used to construct a phylogenetic tree. The analysis showed that DBEs could be clustered into four clades, clade I, clade II, clade III and clade IV, which corresponded to ISAIII, ISAI, ISAII and PUL of maize, respectively (Supplementary Fig. 14). Notably, not all species have the same number of DBE isoforms. This variation in the number of DBE isoforms was closely associated with the number of AGPase, SS and SBE isoforms (Fig. 2). This result suggested that the differential inheritance of DBE genes and lineage-specific expansions was a major component of DBE gene evolution and that the expansion of DBE genes was likely regulated by gene-function balance in starch metabolism (Fig. 9 and Supplementary Fig. 14).

**Figure 10:**
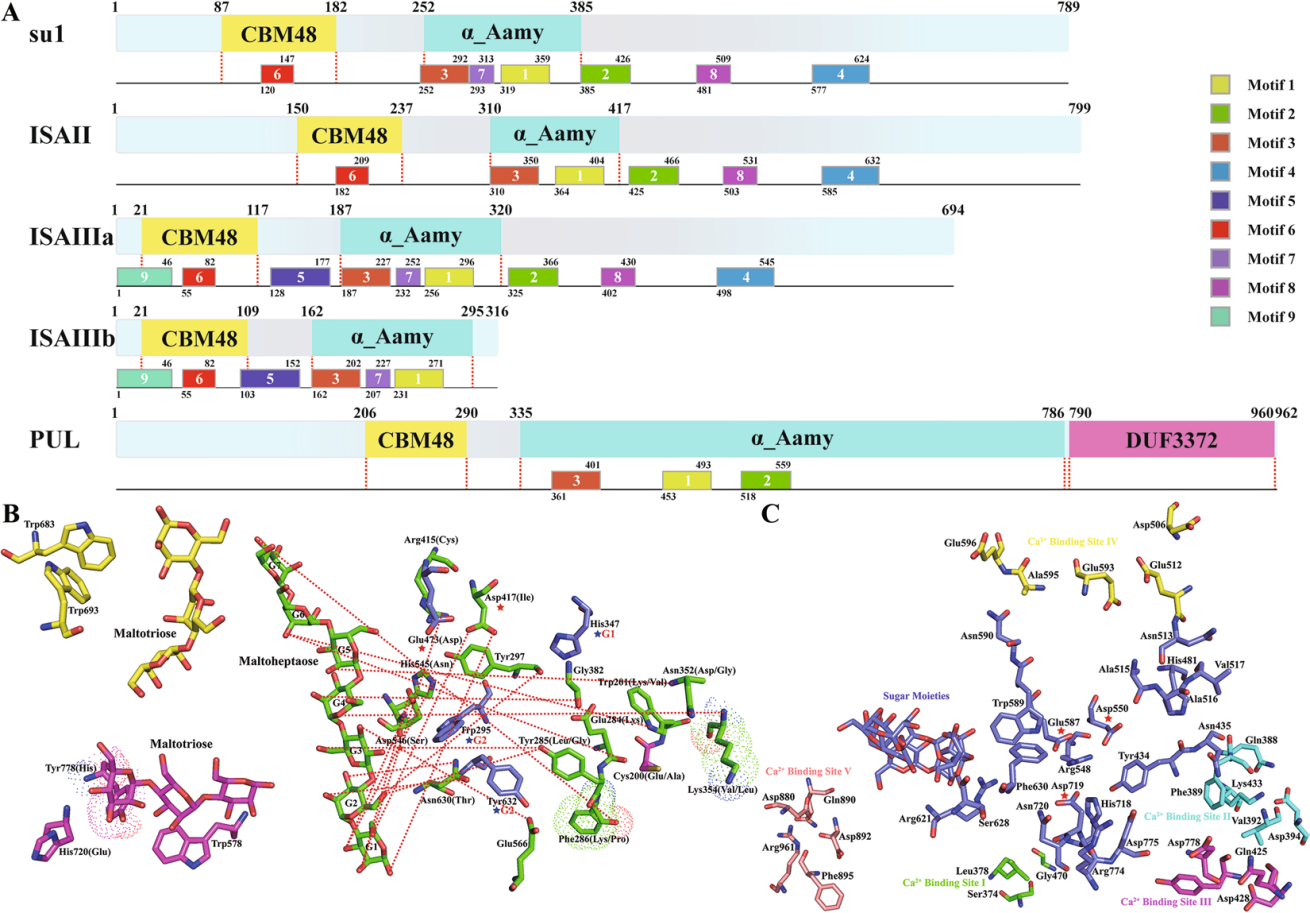
Structural and functional site analysis of maize SBE proteins. Maize is shown as an example. (**A**) Compositions and distributions of domain structures and conserved motifs of DBE proteins are marked and annotated in different colours. (**B**) Stereo view of the active sites of DBE isoforms based on the sequence of su1 and (**C**) PUL. In SBE isoforms, the same site with different amino acids is marked with dots. Different coloured stars and amino acids indicate different functional sites. For the detailed functions of the amino acid sites in SBE and DBE isoforms, please refer to Supplementary Figs 13 and 17.

DBE and SBE share a common modular architecture, but the greatest difference between them is that SBEs usually have an α-amylase C-domain, while isoamylase-type DBE has only a long extension area with a DUF3372 domain in pullulanase-type DBE (Fig. 10A). Further secondary structure analysis of DBE isoforms revealed that the Aamy domain was more enriched in α helixes and β sheets in PUL than in ISA isoforms. As a special PUL domain, the DUF3372 domain contains six α helixes and seven β sheets (Supplementary Fig. 15). Furthermore, the divergence in secondary structures among ISA isoforms is relatively small except for motif 5 and motif 9, which are special motifs in ISAIII, and motif 7, which is shared by both ISAIII and su1 (also named ISAI) (Fig. 10A and Supplementary Fig. 16). These results are consistent with the DBE gene phylogeny and suggest that divergences among ISA isoforms have had little effect on their structures. Remarkably, both ISAs and PUL have unique functional regions and specific structures, indicating that they likely play unique roles in regulating the crystallization and degradation of starch.

To identify subtle differences in DBE isoforms, we aligned the protein sequences of DBE isoforms with those reported for the *Chlamydomonas* ISAI protein (52% identity with maize ISAI) and wheat PUL (74% identity with maize PUL)^34–36^. The analysis showed that the binding and catalytic sites of maltotriose and maltopentaose were not conserved in ISAII and that partial residues were changed in ISAIII (Fig. 10B and Supplementary Fig. 17). Notably, these altered residues may not only change the configuration but may also constrain interactions between active sites and specific substrates. Additionally, five binding sites for calcium ions were found in PUL, and of these, the first to fourth were near the active cleft and the fifth was in the C terminus. These calcium ion binding sites were near the sugar moiety binding sites and catalytic sites, suggesting that calcium ions may, to some extent, affect the interactions between PUL and specific substrates (Fig. 10C).

### Temporal and spatial expression patterns of core genes for starch metabolism

**Figure 11:**
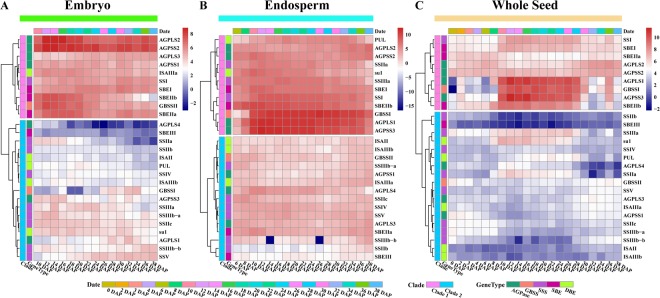
Expression patterns of starch synthesis-related genes during maize embryogenesis and endosperm and kernel development. The embryonic module includes sixteen developmental stages, the endosperm module includes eighteen developmental stages, and the whole seed module includes twenty-one developmental stages. Coloured bars represent the date, cluster and gene symbol. The scale bar shows the normalized RPKM values.

RNA-seq data obtained from multiple materials and organizations and at different developmental stages were used to explore the dynamic expression patterns of core genes related to starch synthesis in maize^37^ (Supplementary Table 2). In embryos of the maize inbred line B73, twenty-seven key genes for starch biosynthesis could be divided into two clusters according to their expression levels, as follows: ten genes with relatively high expression and sixteen with relatively low expression (Fig. 11A). Of these, the high expression genes, including two AGPLSs and two AGPSSs, formed an optimal heterotetramer that could initiate starch biosynthesis in the embryo. The other six genes were mainly responsible for the synthesis and modification of amylose and short amylopectin. These results suggested that amylose and short amylopectin were likely the main products during embryo development.

In the endosperm of maize inbred line B73, seventeen developmental stages were selected to analyse the expression patterns of key regulatory genes in starch synthesis. The results showed that twenty-seven of the expressed genes could be further divided into a cluster of fifteen relatively low-expressing genes and a cluster of twelve relatively high-expressing genes (Fig. 11B). Unlike what was observed in embryos, AGPLS1 and AGPSS3 were expressed at high levels in endosperm, in which they replaced AGPLS3 and AGPSS1 to form an optimal heterotetramer that could initiate starch biosynthesis (Fig. 11B). In addition, GBSSI rather than GBSSII was expressed at high levels in endosperm (Fig. 11B). This observation is consistent with previous studies of the early stages of endosperm development^38^ (Supplementary Fig. 18 and Supplementary Table S2). Notably, while AGPLS4, GBSSII, SSV and SBEIIa were expressed at high levels in the endosperm of maize hybrid SD609, they were expressed at low levels in the endosperm of maize inbred line B73 (Fig. 11 and Supplementary Fig. 18). Therefore, differences in starch synthesis in different materials is likely caused by changes in the expression levels of specific genes involved in the process of starch synthesis.

Twenty-seven core genes evaluated at twenty-one developmental stages in whole seeds of maize inbred line B73 were also divided into two clusters based on gene expression levels (Fig. 11C). In particular, we found that the expression patterns of these genes were different from those observed in embryos and endosperm in co-expression modules and an evaluation of fluctuations in the characteristics of specific gene expression patterns during seed growth. For example, AGPLS1, AGPSS3, GBSSI, SSI, SSIIa, SSIIIa, SBEI, SBEIIb and SU1 were expressed at higher levels from approximately 10 days after pollination (DAP) to 30 DAP than during the early and late phases of whole-seed development. Additionally, they displayed two transition points resulting in an up-down expression pattern. This feature may be closely related to the process of seed cell development. The period from 0–10 DAP is the crucial time for cell proliferation and differentiation; while approximately 10–30 DAP, a period of rapid embryo and endosperm growth, is the crucial time for grain filling and yield formation^38–42^. Subsequently, the starch synthesis rate decreases and switches to a drying-out period, during which genes such as SBEIIa, AGPSS1 exhibit a down-up expression pattern throughout the process of seed development (Fig. 11C). These fluctuating expression patterns indicate that the core genes for starch synthesis likely regulate the dominant functions of starch metabolism via these expression transitions.

## Discussion

Starch biosynthesis is a highly regulated metabolic process that requires the coordinated activities of multiple enzymes, and most of the enzymes involved in these catalytic reactions are the same between amyloplasts and chloroplasts, as shown in Fig. 1. In green plants, the starch biosynthesis pathway involves a complex network of genes, most of which are members of large multigene families with multiple isoforms. However, the starch synthesis-related enzyme isoforms have not yet been identified and classified in some plants. To date, homology hybridization and PCR screening are two main strategies for screening starch biosynthesis-related genes, but because these screening processes are based on conserved known gene sequences in addition to protein isolation, purification and sequencing, they may fail to identify novel isoforms of starch biosynthesis-related enzymes that have low sequence similarities with other known genes involved in starch biosynthesis or they may fail to purify all isozymes because they may have extremely similar activities and molecular weights across green plants. Recently, the development of next-generation high-throughput sequencing technologies has provided a robust tool for using full-length cDNAs to map and quantify the genome in many plant species. This has provided unique opportunities to use genome-wide screening to study starch synthesis-related enzyme families. Moreover, combining DNA/protein sequence information from sequenced genomes with molecular biology experiments is a good strategy for isolating and verifying new genes in different plant species.

Initial starch biosynthesis can be traced back to plastid endosymbiosis in photosynthetic eukaryotes. A primary endosymbiotic event occurred in a heterotrophic eukaryotic cell that internalized a cyanobacterial cell, bringing plastids into eukaryotes, thereby rendering them able to perform oxygenic photosynthesis during the continuous evolution of cyanobacterial endosymbionts (cyanobionts)^43,44^. Moreover, protein-targeting machinery in the cytosol of this evolving plastid appeared and retargeted the organelle for transhipment of the remaining genes and their products. Additionally, subsequent to the endosymbiosis of the plastid, the cyanobiont polysaccharide storage metabolism was reconstructed to perform starch metabolism. Additionally, monophyletic Archaeplastida emerged and spawned three Archaeplastida lineages: Glaucophyta (glaucophytes), Rhodophyceae (red algae) and Chloroplastidae (green algae and all land plants)^14,45^. In particular, glaucophytes and red algae produce and store starch in the cytoplasm, whereas green algae and all land plants perform starch biosynthesis and then store starches in the plastid compartment^46^.

In this paper, a phylogenetic analysis suggested that the first duplication in the AGPLS family occurred earlier than the duplication in the AGPSS family, and there were more gene duplications in AGPLS than in AGPSS (Fig. 2 and Supplementary Fig. 1). In particular, AGPase is involved in the synthesis of starch, during which it acts as a heterotetramer in angiosperms and green algae. Nevertheless, there is a mismatch in the numbers of AGPLS subunits in these combinations (Supplementary Fig. 1), suggesting that AGPLS and AGPSS may have been exposed to different selection pressures over time and that the composition of these heterotetramers during starch biosynthesis may differ among different species or be dynamic across different developmental stages in the same plant species. Importantly, the two AGPLSs or the two AGPSSs in the heterotetramer perform complementary rather than redundant functions^24,47^. Furthermore, the spatio-temporal expression profiles of AGPase gene subunits also indicate that the potential combinations of AGPLS and AGPSS are different in different locations during starch synthesis (Fig. 11). AGPase acts as a transporter of glucose during starch synthesis, and its constituent components are key factors that limit starch accumulation to specific locations^5^.

The phylogenetic relationships among SSs showed that there were more gene duplications in SSII and SSIII than in other SSSs and that SSIV and SSV are phylogenetically closest. Moreover, there are more structural similarities among SSI, SSII and GBSS than among SSIII, SSIV and SSV (Fig. 6). These divergences between SS isoforms are likely related to their genetic origin and the occurrence of gene duplications. Previous studies have supported the idea that GBSS was acquired through endosymbiotic gene transfer from a symbiont^14^. SSI and SSII, similar to GBSS, evolved from the symbiont, while the ancestor of SSIII-SSV was transmitted via lateral gene transfer from intracellular chlamydial pathogens, and two subsequent duplications resulted in three clades encoding SSIII, SSIV and SSV^10,14,23^. In addition, we infer that the deletion or insertion of nucleotide fragments or sites could potentially cause functional differences in SS isoforms after gene duplication, leading to their sub-functionalization or neo-functionalization. Our phylogenetic analysis of SBEs showed that there is an obvious difference in isoform numbers among plant species and that the SBEII class is subdivided into two or more distinct gene clusters in some green plants (Supplementary Fig. 10). These results are likely related to the main types of starches present in some plant species. For example, SBEI has a preference for amylose as a substrate and transfers relatively longer glucan chains (up to DP 30, with the majority being DP 10–13), while SBEII isoforms transfer shorter chains (DP 6–14) and prefer amylopectin as a substrate^48–50^.

During starch metabolism, SSI and SSII isoforms and either SBEIIa or SBEIIb form a trimeric complex to regulate the starch metabolic rhythm. For example, in maize, this trimeric complex generally consists of SSI, SSIIa and SBEIIb^51^. Additionally, a new stromal complex involving SSI, SSIIa, SBEI, SBEIIa and phosphorylase (PHO1) was found in the amylose extender (ae1.1) mutant, and SBEIIb deficiencies have been shown to affect SSI and SBEI binding to starch granules^52,53^. Moreover, subsequent experiments showed that PHO1 interacts with SBEI or SBEIIb^54^. The outcome of the amylose extender (ae1.2) mutation experiment also showed that SSI, SSIIa, SBEI and SBEIIb form another multiple enzyme complex, and there were obvious differences in amylose content and granule size between two *ae* mutations from two near-isogenic maize lines^55^. Furthermore, a high molecular weight, multiple enzyme complex composed of SSIIa, SSIII, SBEIIa and SBEIIb was found in maize and was shown to further assemble into a 670-kD complex by interacting with pyruvate orthophosphate dikinase (PPDK) and the sucrose synthase isoform SUS-SH1^33^. A lack of SSIIIa function, as in the maize dull mutation, caused a decrease in the activity of SBEIIa and SSII. Researchers were also able to use SSIII to co-precipitate the small subunit of AGPase encoded by bt2, and the large subunit of AGPase encoded by sh2 was recovered from an SSIIIHD affinity column^27,56^. In particular, a temporal expression pattern analysis of genes in the 670-kD complex revealed that while the expression levels of SSIIa, SSI and SBEIIb were similar in maize endosperm, they were out of sync in embryos and whole seeds (Fig. 11). Thus, we propose that the forms this complex takes in different structures are likely to be closely related to the expression patterns of these genes. For instance, a compatible combination composed of AGPLSs and AGPSSs is selectively expressed at high levels in different tissues (Fig. 11).

**Figure 12:**
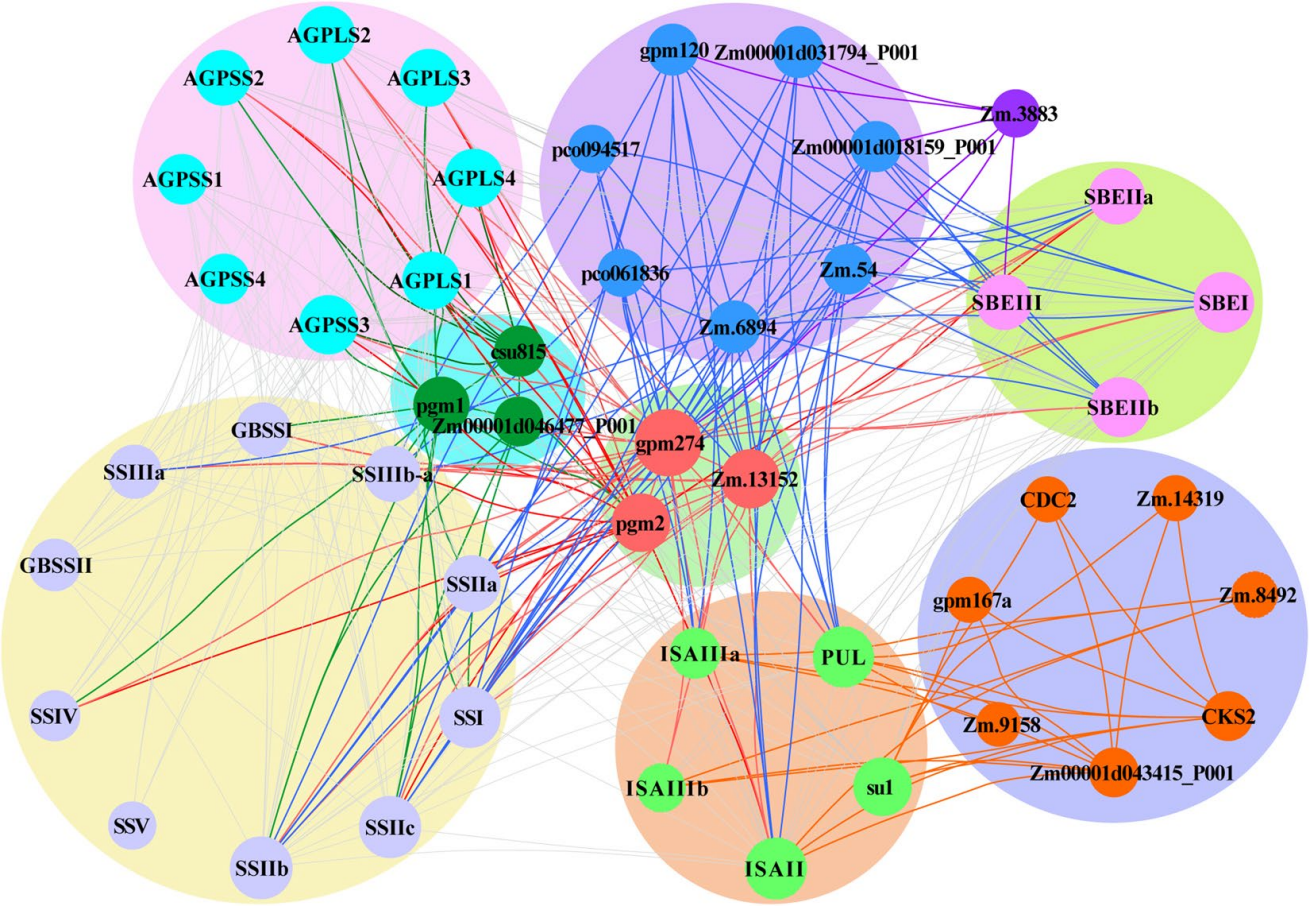
Interaction network of starch synthesis-related enzymes. Interactions among SS and AGPase subunits and SBE and DBE isoforms are shown as grey lines. Interactions between starch synthesis-related enzymes and other proteins are shown as specific coloured lines.

A previous study demonstrated that SSIII isoforms and SSIV were involved in starch granule initiation, whereas SSIII and SSIV double-mutant plants could not accumulate starch^57^. Nevertheless, these proteins were not completely equivalent in regulating the synthesis of starch granules because SSIII but not SSIV was able to use ADP-glucose as a substrate to synthesize linear glucans without other primers^57–59^. The functional divergence between these isoforms was not only related to their active sites and gene expression patterns but may also have been affected by other interacting proteins. An analysis of potential interrelationships between proteins showed that core proteins involved in starch synthesis might interact directly or communicate with each other via other proteins (Fig. 12). Moreover, these starch synthases all have specific interaction proteins, except for certain proteins that form homopolymers.

A phylogenetic analysis of DBEs suggested that there is no significant difference in the numbers of ISAI, ISAII, ISAIII and PUL in green plants except for the structural difference between PUL and ISAI-ISAIII (Fig. 10 and Supplementary Fig. 14). Mutation experiments performed in ISAs (ISAI and ISAII) and SSIIIa showed that SSIIIa could compensate for a lack of ISAII in the ISAI/ISAII heteromer and coordinate the ability of the ISAI homomer to regulate normal starch crystallization and restrict phytoglycogen accumulation^7^. Moreover, a study of a double mutant for ISAI and PUL demonstrated that the function of PUL partially overlapped with that of ISAI and that the contribution of PUL to amylopectin trimming was much weaker than that of ISAI^60^. However, the details of the mechanisms underlying coordination between ISAI and PUL during the regulation of normal starch crystallization remains unclear. Nevertheless, our analysis of protein-protein interaction networks indicated that su1 remained in contact with PUL via two hub proteins, CKS2 and a protein encoded by the *Zm00001d043415* gene (Fig. 12). These results provide novel insight that increases our understanding of the detailed interrelationships between the core genes involved in starch synthesis.

## Methods

### Data collection

In the present study, the sequenced genomes and corresponding proteomes of 74 plant species were used to establish an initial data set., These included twelve species from Fabales, ten from Brassicales, three from Malvales, two citrus species, six from Malpighiales, six aster species, two from Caryophyllales, twelve from Graminales, two from Arecales, three bryophytes, nine chlorophytes, and single genomes and proteomes from Myrtales (*Eucalyptus grandis*), Vitales (*Vitis vinifera Genoscope*), Ranunculales (*Aquilegia coerulea*), Bromeliaceae (*Ananas comosus*), Zingiberales (*Musa acuminata*), Amborellales (*Amborella trichopoda*) and Selaginellales (*Selaginella moellendorffii*). The phylogeny of these species is provided in Supplementary Table 1. Starch synthesis-related gene expression data were obtained from examined RNA-seq data for the maize (*Zea mays*. L) hybrid Shandan 609 and the inbred line B73, which were derived from our previously published (BioProject accession number PRJNA299361) and openly published papers (The National Center for Biotechnology Information Sequence Read Archive accession number SRP037559 and GenBank data library)^37,38^.

### Sequence retrieval

Candidate AGPase, SS, SBE and DBE genes were initially identified using HMMER3.0 with default settings (domain signature NTP_transferase for AGPase; GT1, GT5 and CBM25 for SS; α_Aamy, α_Aamy_C and CBM48 for SBE; and α_Aamy, CBM48 and DUF3372) for each of the 74 proteome data sets^61^. To search for potential AGPase, SS, SBE and DBE genes, the corresponding amino acid sequences acquired in the previous step were used as queries to run a BLASTp search against the proteomes of 74 species in Phytozome with default settings (version 12.1; http://www.phytozome.net/). All hits obtained using the PFAM (http:// pfam.xfam.org/search), CDD (http://www.ncbi.nlm.nih.gov/Structure/cdd/wrpsb.cgi), and SMART (http://smart.embl-heidelberg.de/) databases were further verified. Sequences that did not have a detectable domain or a threshold E-value of less than 1e-10 were excluded. Only the longest transcript was retained when two or more transcripts were identified from the alternative splicing of a gene. Finally, the genes verified by the above steps were used in this study.

### Phylogenetic analysis

Amino acid sequences of all identified candidate AGPase, SS, SBE and DBE genes were aligned in ClustalW v2.1 with default settings^62^. The obtained alignments were then manually corrected in MEGA 7.0 software^63^. Phylogenetic trees were constructed using the maximum likelihood method with PhyML 3.0, the substitution model was assessed with the Akaike information criterion, and the reliability of internal nodes was evaluated by calculating Shimodaira-Hasegawa approximate likelihood ratio test (SH-aLRT) values^64^. This evaluation method has been shown to be an accurate, powerful and robust tool for processing large data sets^65^. Finally, the phylogenetic tree was annotated and visualized using iTOL v3^66^.

### Motif analysis

All amino acid sequences of maize AGPase, SS, SBE and DBE genes were analysed with MEME (v4.11.4) to discover novel conserved patterns (http://meme-suite.org/tools/meme). The following parameters were used: repetitions per sequence = zero or one occurrence per sequence and the number of motifs selected was based on an E-value less than 10e-10 and other parameters as default settings.

### Structural model building

The amino acid sequences of maize AGPase, SS, SBE and DBE isoforms were retrieved and submitted to the SWISS-MODEL (https://swissmodel.expasy.org/) and RCSBPDB (http://www.rcsb.org/pdb/home/home.do) databases to search for the best reference model. Then, the PDB files corresponding to each starch-related enzyme isoform were analysed, visualized, and edited with PyMOL (0.99rc6) software (http://www.pymol.org/).

### Gene expression and protein interaction analysis

RNA-seq data were obtained from an article that we previously published and from openly published papers and corresponding libraries that were produced using the methods described in these papers^37,38^. The expression levels of the four types of starch metabolism-related enzyme isoform-encoding genes were computed in RPKM (reads per kilobase of exon per million mapped reads) on maize gene models. Then, gene expression values were normalized (log2(expression value of genes)) for hierarchical clustering, and the complete linkage method as well as the Euclidean distance measure were used for hierarchical clustering of gene expression profiles with the R function hclust. Additionally, protein sequences of multiple enzyme isoforms were retrieved for protein-protein interaction analysis in string software (v10.5) (https://string-db.org/). The protein-protein interaction network was edited with Cytoscape software (version 3.4.0; http://www.cytoscape.org/download.php)^67^. All statistical analyses and drawings were performed using the R language (http://www.r-project.org).

## Electronic supplementary material

Supplementary Information

Supplementary Dataset 1

Supplementary Dataset 2
