# Supplementary material for: Evolutionary, structural and expression analysis of core genes involved in starch synthesis
Source: Sci Rep. 2018 Aug 24;8:12736. doi: 10.1038/s41598-018-30411-y (PMC6109180; doi:10.1038/s41598-018-30411-y)
Supplement: Supplementary file 1 — Supplementary Information [file 41598_2018_30411_MOESM1_ESM.docx]

**Evolutionary,** **structural and expression analysis of core genes involved in starch synthesis**

**Jianzhou Qu,^1,2^ Shutu Xu,^1,2^ Zhengquan Zhang,^1,2^ Guangzhou Chen,^1,2^ Yuyue Zhong,^1,2^ Linsan Liu,^1,2^ Renhe Zhang,^1,2^ Jiquan Xue,^1,2,*^ Dongwei Guo,^1,2,*^**

**^1^**The key Laboratory of Biology and Genetics Improvement of Maize in Arid Area of Northwest Region, Ministry of Agriculture, College of Agronomy, Northwest A&F University, Yangling 712100, Shaanxi, China;

**^2^**Maize Engineering Technology Research Centre of Shaanxi Province, Yangling 712100, Shaanxi, China

* Corresponding author: xjq2934@163.com (J.Q.X.); gdwei1973@126.com (D.W.G)

**List of Supplementary Information**

**Supplementary Table 1** Protein sequence information for 74 plant species.

**Supplementary Table 2** Gene expression levels of core genes involved in starch synthesis.

**Supplementary Fig. 1** Phylogenetic tree of AGPases.

**Supplementary Fig. 2** Motif logo of AGPase subunits.

**Supplementary Fig. 3** Three-dimensional modelled structures of AGPase subunits in maize.

**Supplementary Fig. 4** Active sites of AGPase subunits.

**Supplementary Fig. 5** Phylogenetic tree of SSs.

**Supplementary Fig. 6** Cartoon representing the secondary structural organization of SS isoforms.

**Supplementary Fig. 7** Motif logo of SS isoforms.

**Supplementary Fig. 8** Protein sequence alignment of maize SS isoforms, barley SSI and *Escherichia coli* glycogen synthase.

**Supplementary Fig. 9** Protein sequence alignment of maize GBSS isoforms, rice GBSSI, *A. tumefaciens* glycogen synthase and *Escherichia coli* glycogen synthase.

**Supplementary Fig. 10** Phylogenetic tree of SBEs.

**Supplementary Fig. 11** Three-dimensional modelled structures of SBE isoforms.

**Supplementary Fig. 12** Motif logo of SBE isoforms.

**Supplementary Fig. 13** Active site alignment of maize SBE isoforms, rice BEI and *Escherichia coli* glycogen branching enzyme.

**Supplementary Fig. 14** Phylogenetic tree of DBEs.

**Supplementary Fig. 15** Cartoon representing the secondary structural organizations of DBE isoforms.

**Supplementary Fig. 16** Motif logo of DBE isoforms.

**Supplementary Fig. 17** Active site alignment of maize DBE isoforms, *Chlamydomonas* ISA1, PUL of *K. pneumoniae* and Barley.

**Supplementary Fig. 18** Expression patterns of starch synthesis-related genes in endosperm.


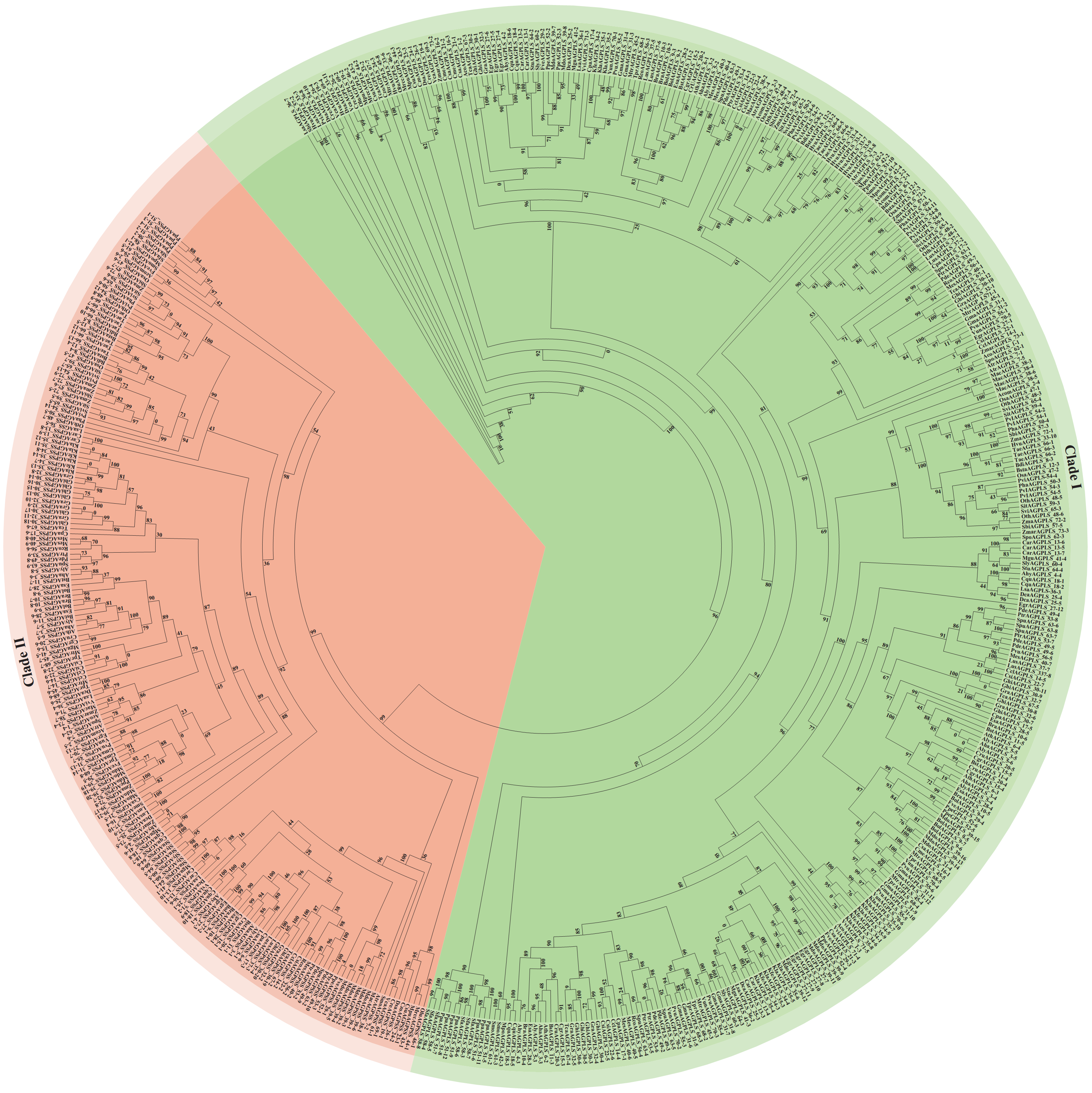
**Supplementary Fig. 1 Phylogenetic tree of AGPases.** The protein sequences of AGPase in 73 plant species were divided into two clades, represented as AGPLS and AGPSS.


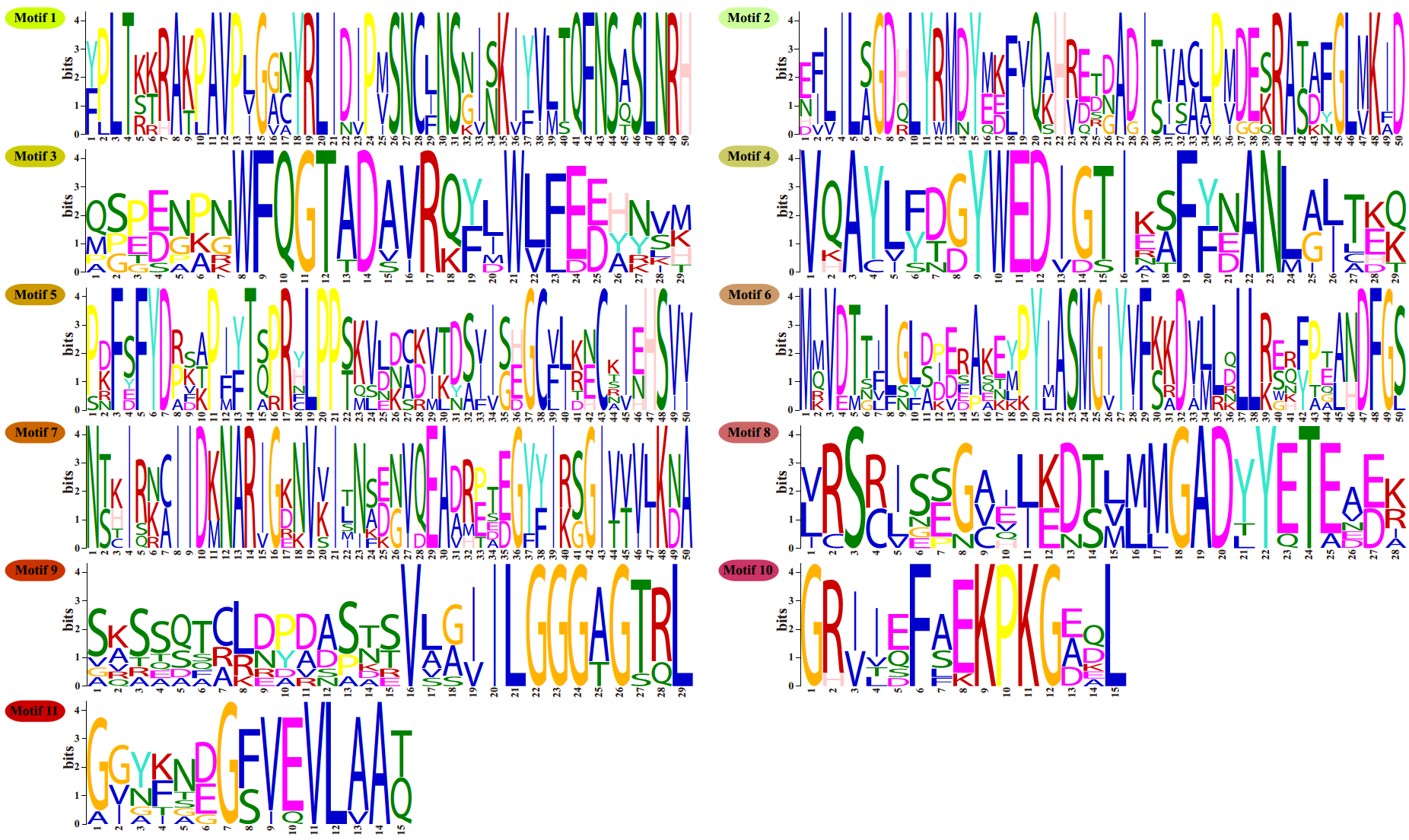
**
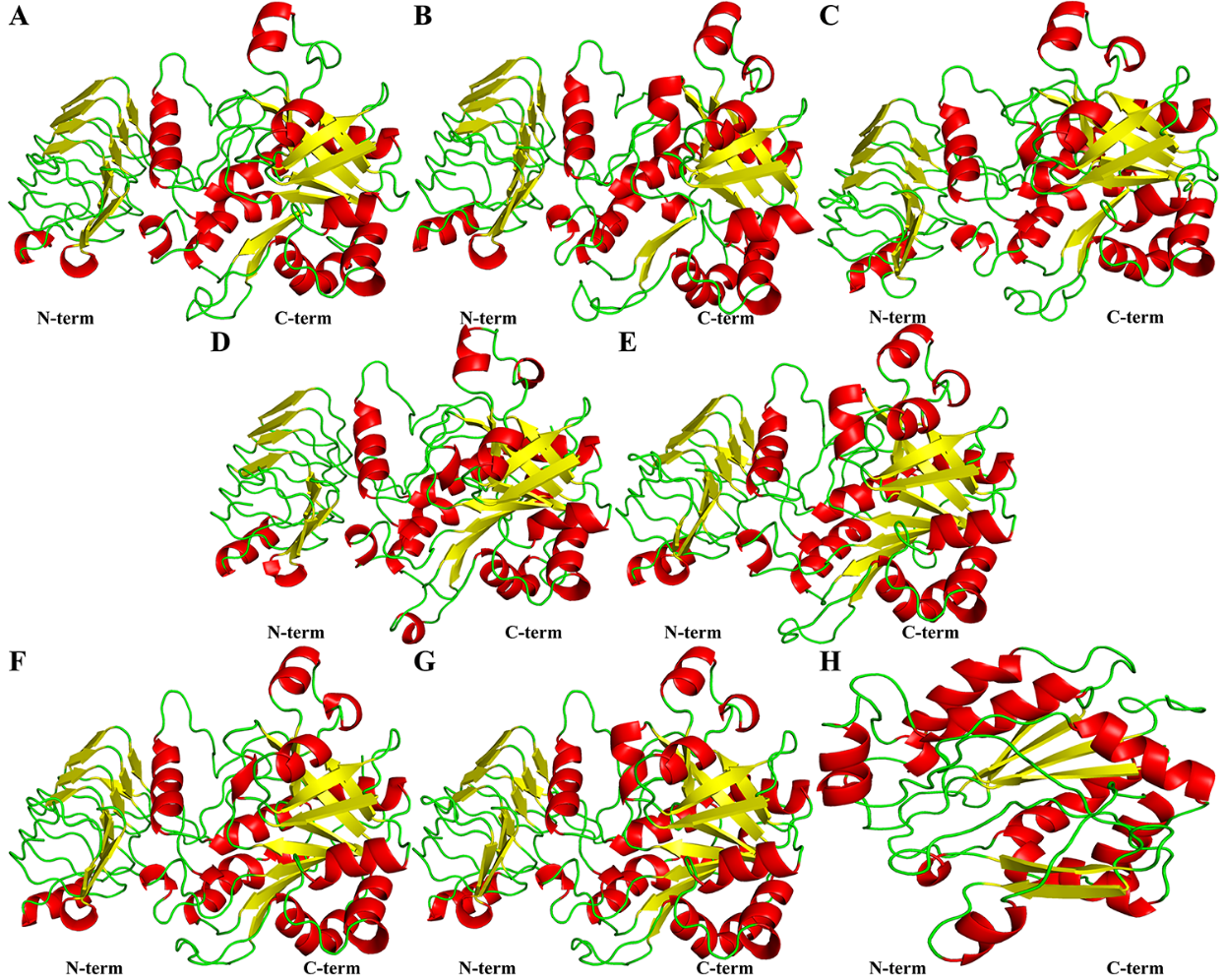
Supplementary Fig. 2 Motif logo of AGPase subunits.**

**Supplementary Fig. 3 Three-dimensional modelled structures of AGPase subunits in maize.** Α, B, C, D, E, F, G and H show the crystal structures of AGPLS1-4 and AGPSS1-4, respectively. In the cartoon structure of these AGPase subunits, an α-helix is coloured red, and a β-fold is coloured yellow.

**
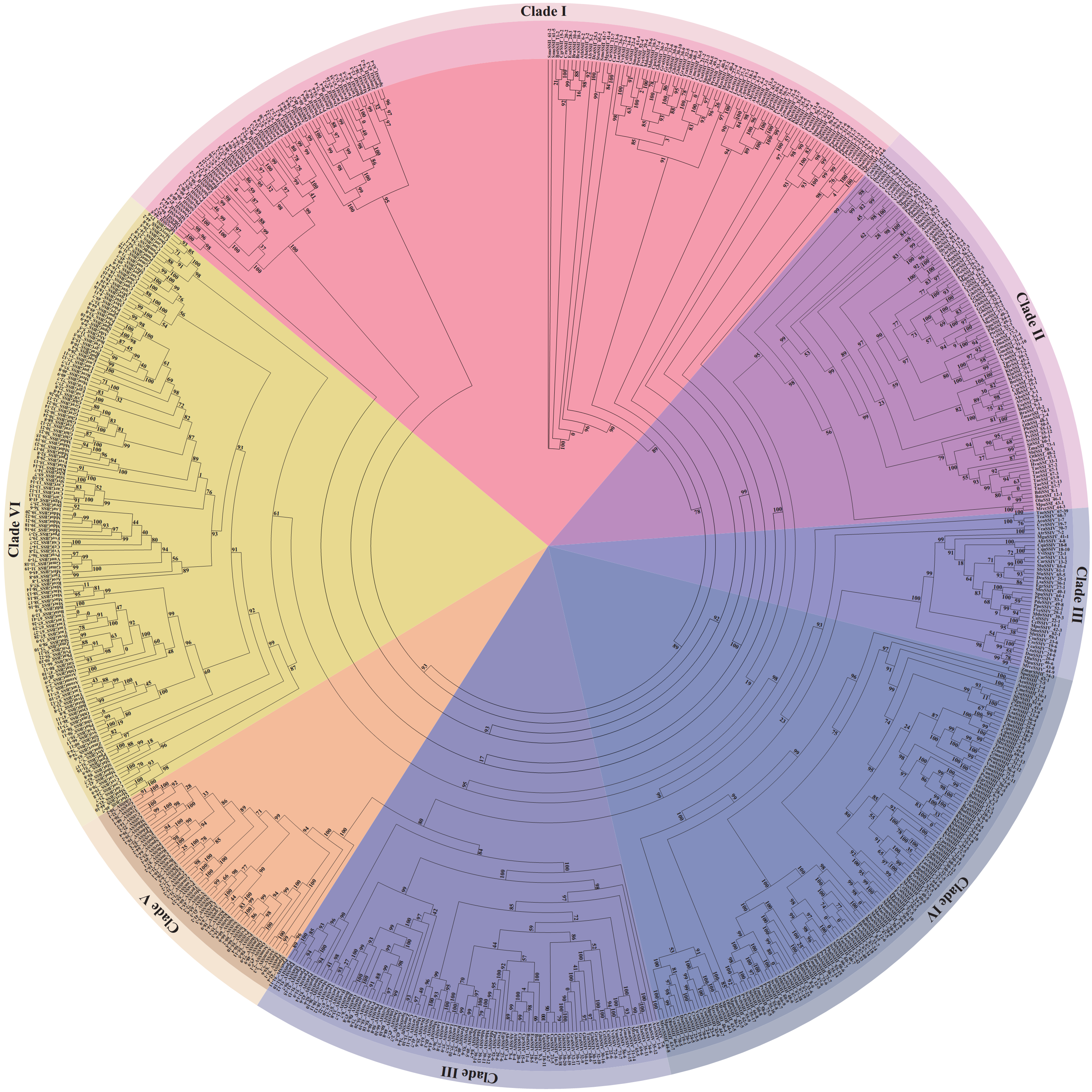

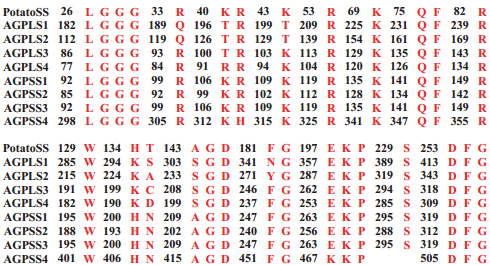
Supplementary Fig. 4 Active sites of AGPase subunits.** Red indicates amino acid residues that represent active sites in AGPase subunits.


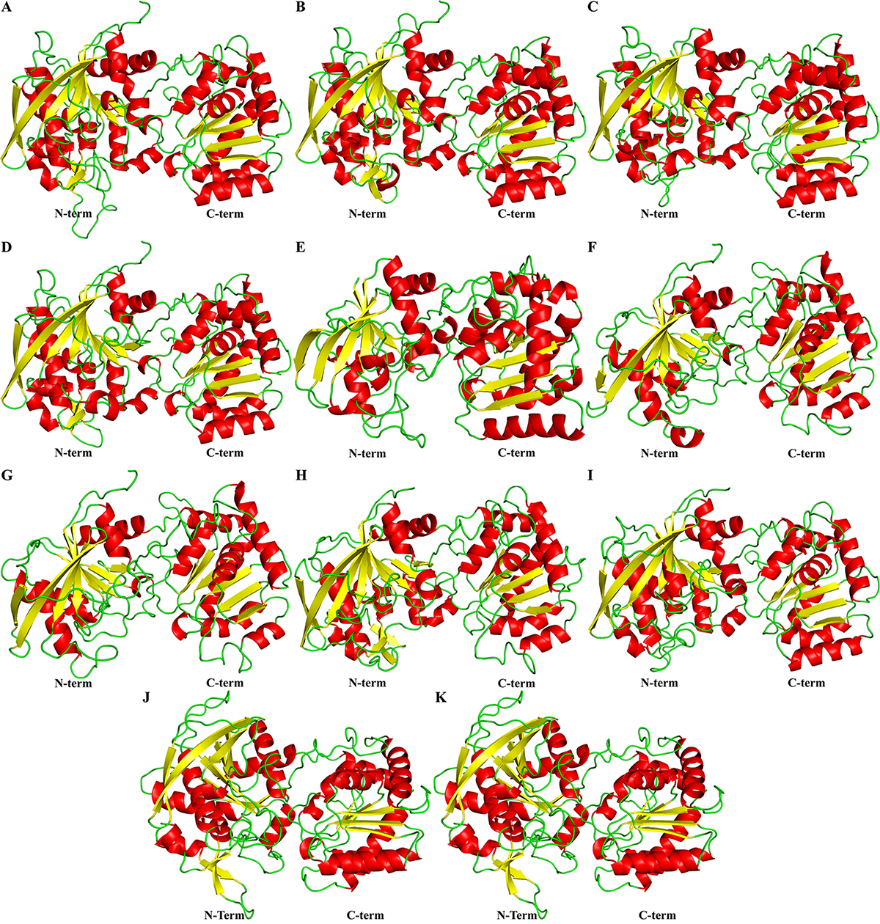
**Supplementary Fig. 5 Phylogenetic tree of SSs.** The protein sequences of SS in 74 plant species were divided into six clades. Clade I, clade II, clade III, clade IV, clade V and clade VI represent SSII, SSI, SSIV, SSIII, SSV and GBSS, respectively.

**Supplementary Fig. 6 Cartoon representing the secondary structural organization of SS isoforms.** Α-K show the overall structures of SSI, SSIIa, SSIIb, SSIIc, SSIIIa, SSIIIb-a, SSIIIb-b, SSIV, SSV, GBSSI and GBSSIIa, respectively. Meanwhile, α-helixes are coloured red, and β-folds are coloured yellow.

**
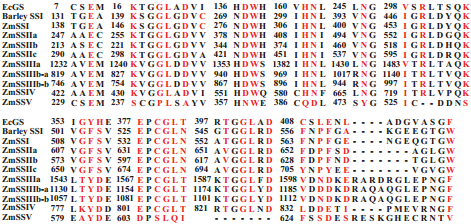

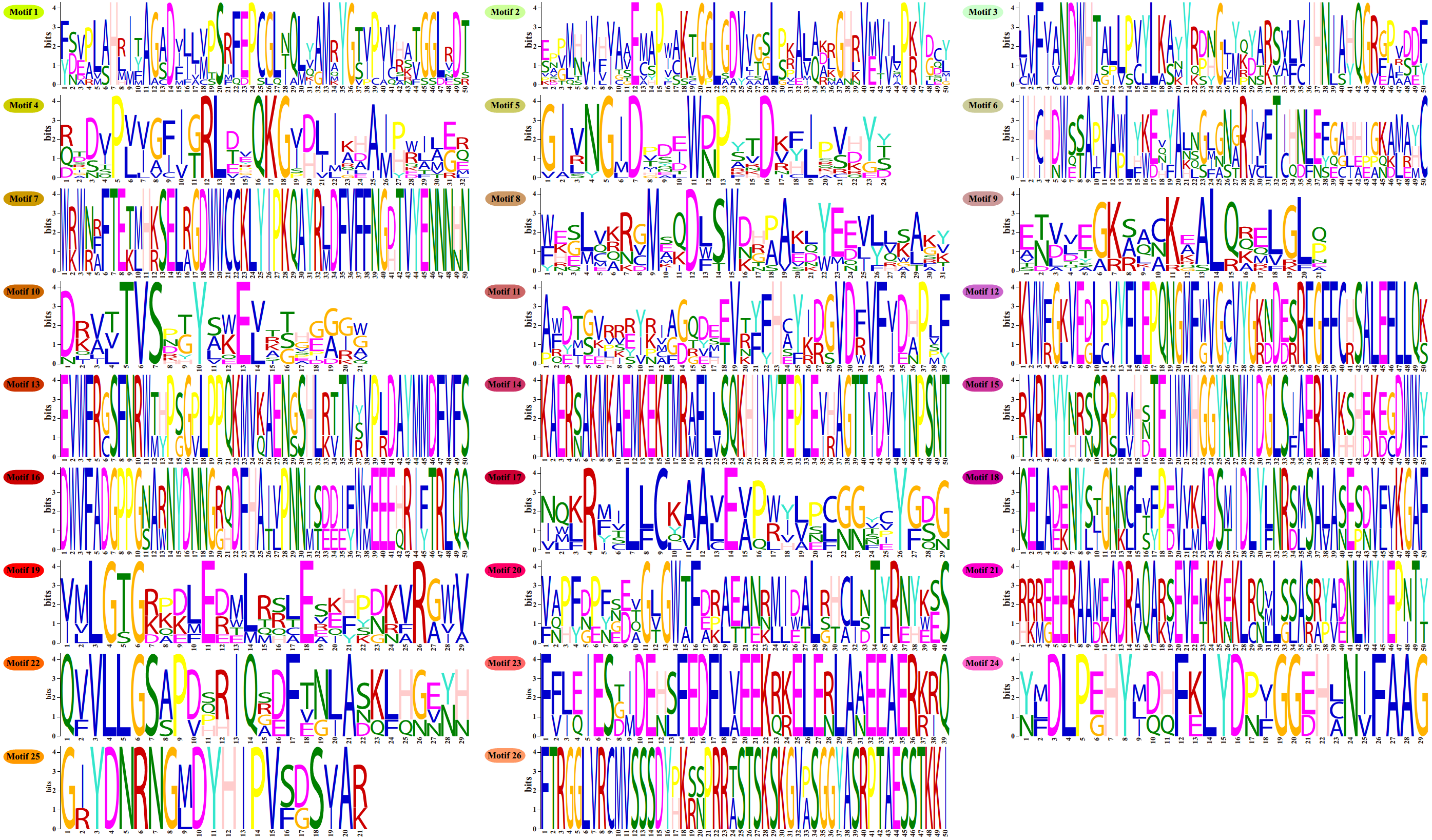
Supplementary Fig. 7 Motif logo of SS isoforms.**

**Supplementary Fig. 8 Protein sequence alignment of maize SS isoforms, barley SSI and *Escherichia coli* glycogen synthase.** Red indicates amino acid residues that represent active sites in maize AGPase isoforms.

**
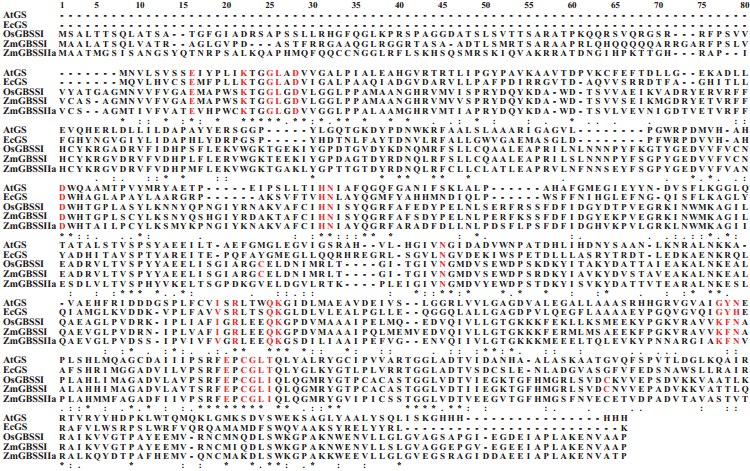
**
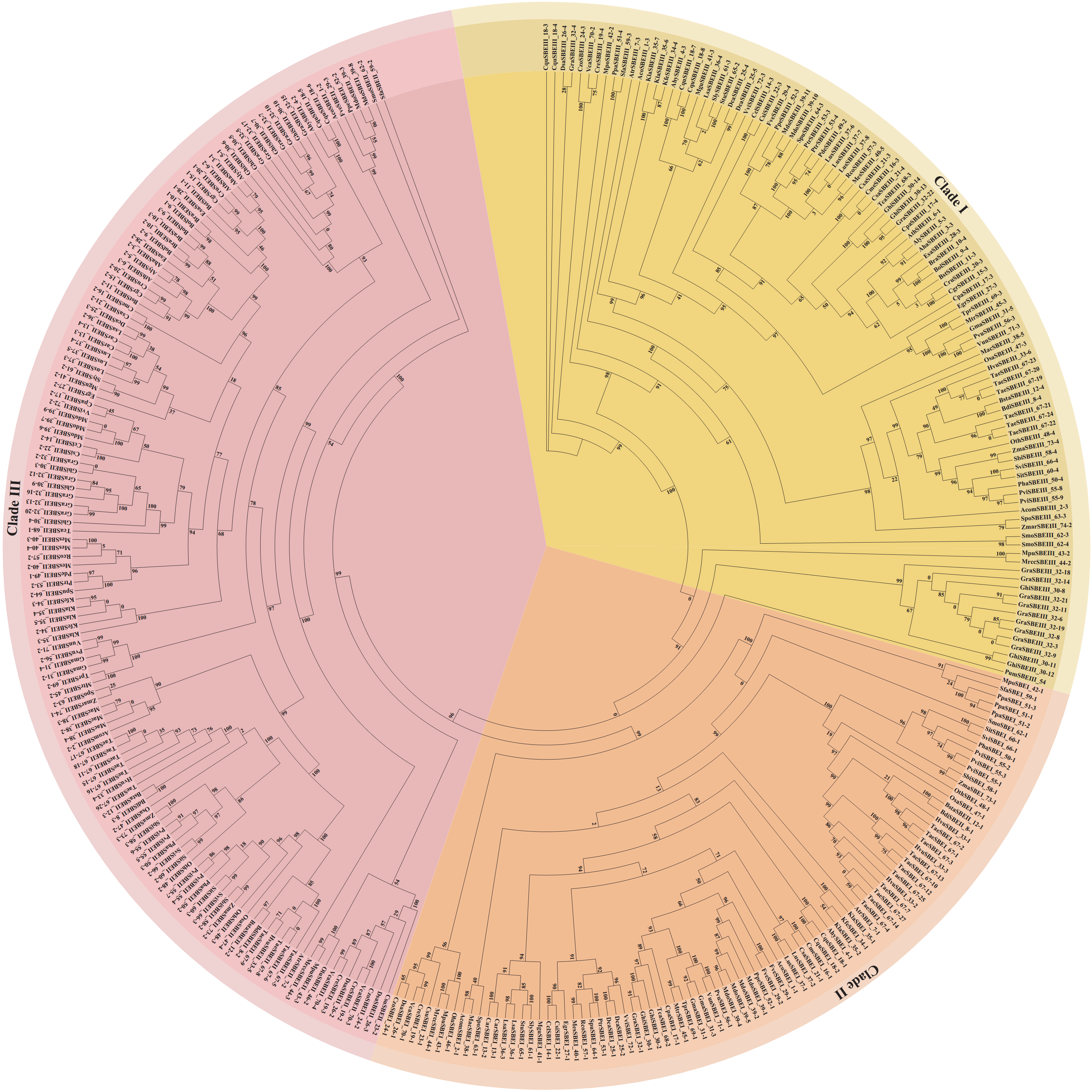
**Supplementary Fig. 9 Protein sequence alignment of maize GBSS isoforms, rice GBSSI, *A. tumefaciens* glycogen synthase and *Escherichia coli* glycogen synthase.** Red indicates amino acid residues that represent active sites in maize GBSS isoforms.


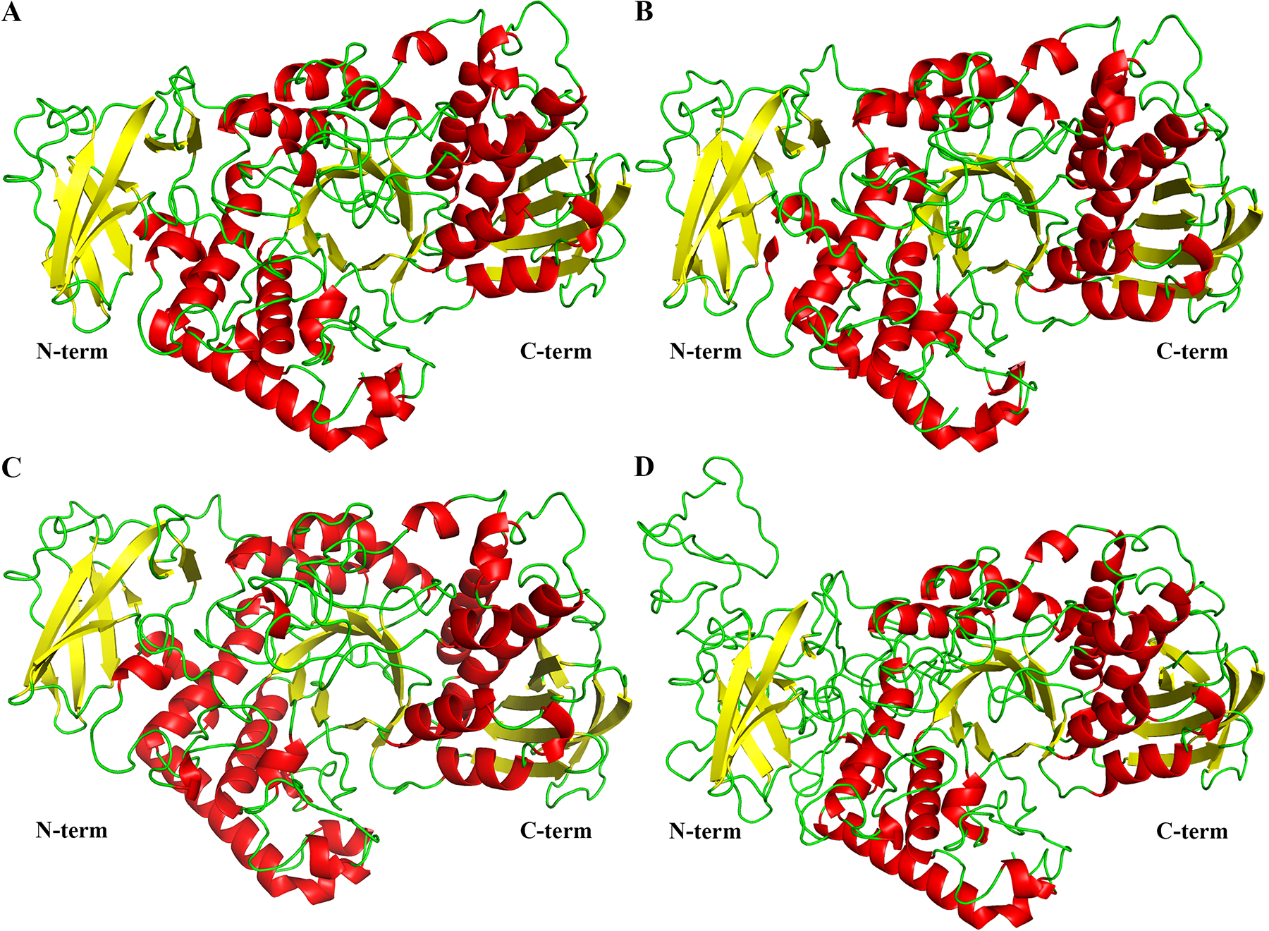
**Supplementary Fig. 10 Phylogenetic tree of SBEs.** The protein sequences of SBE in 74 plant species were divided into three clades. Clade I, clade II and clade III represent SBEI, SBEII and SBEIII, respectively.

**Supplementary Fig. 11 Three-dimensional modelled structures of SBE isoforms.**


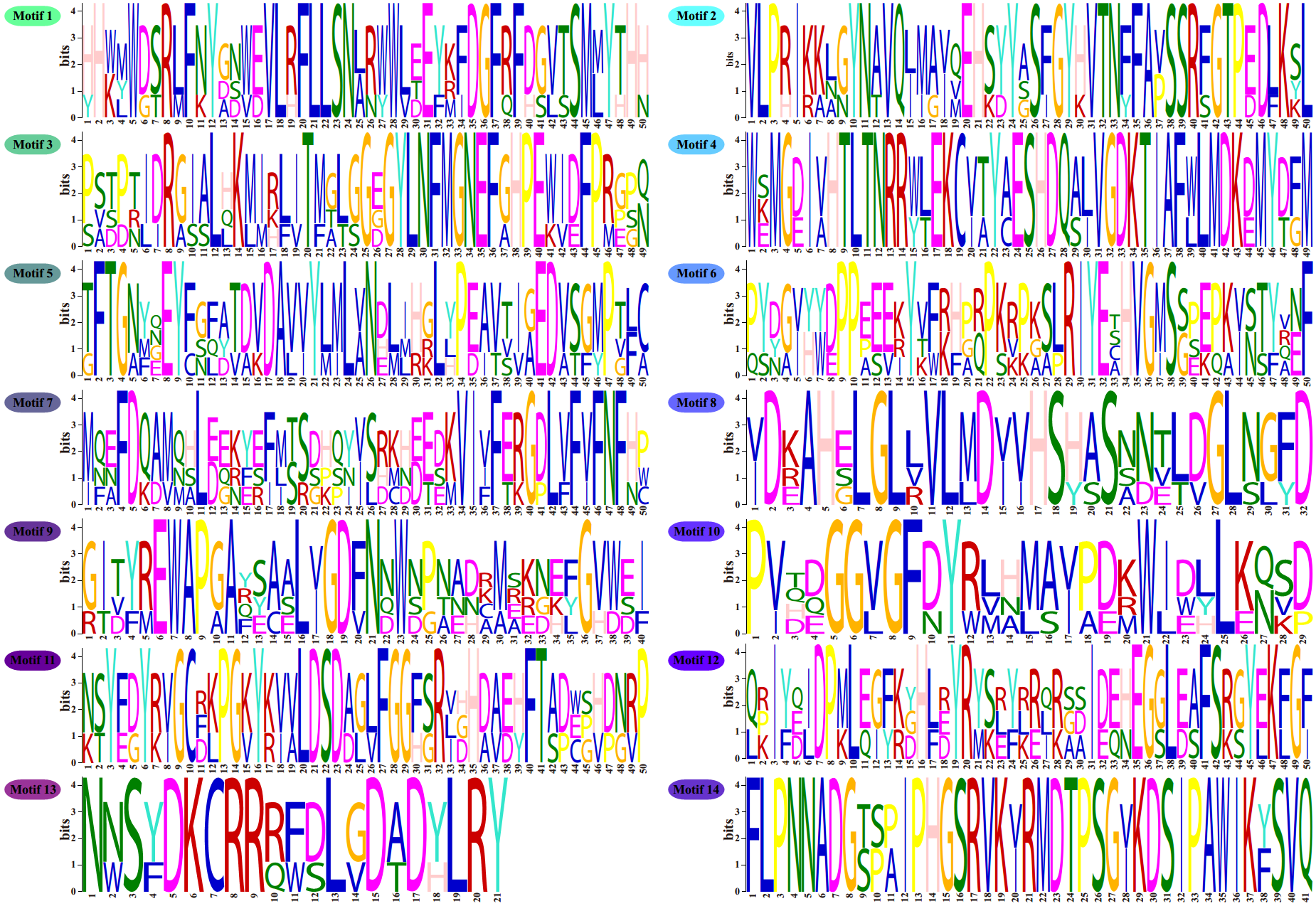
A-D show the secondary structures of SBEI, SBEIIa, SBEIIb and SBEIII, respectively. Additionally, α-helixes are coloured red, and β-folds are coloured yellow.


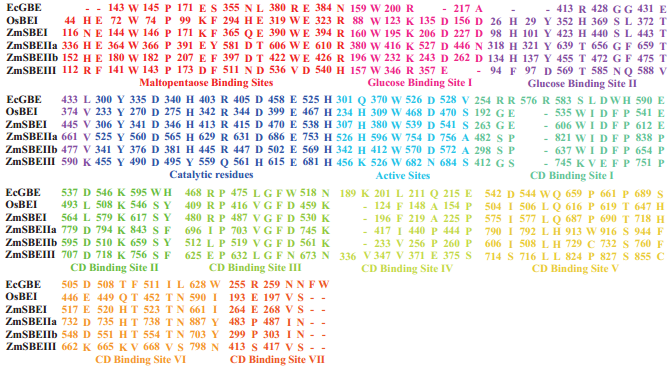
**Supplementary Fig. 12 Motif logo of SBE isoforms.**


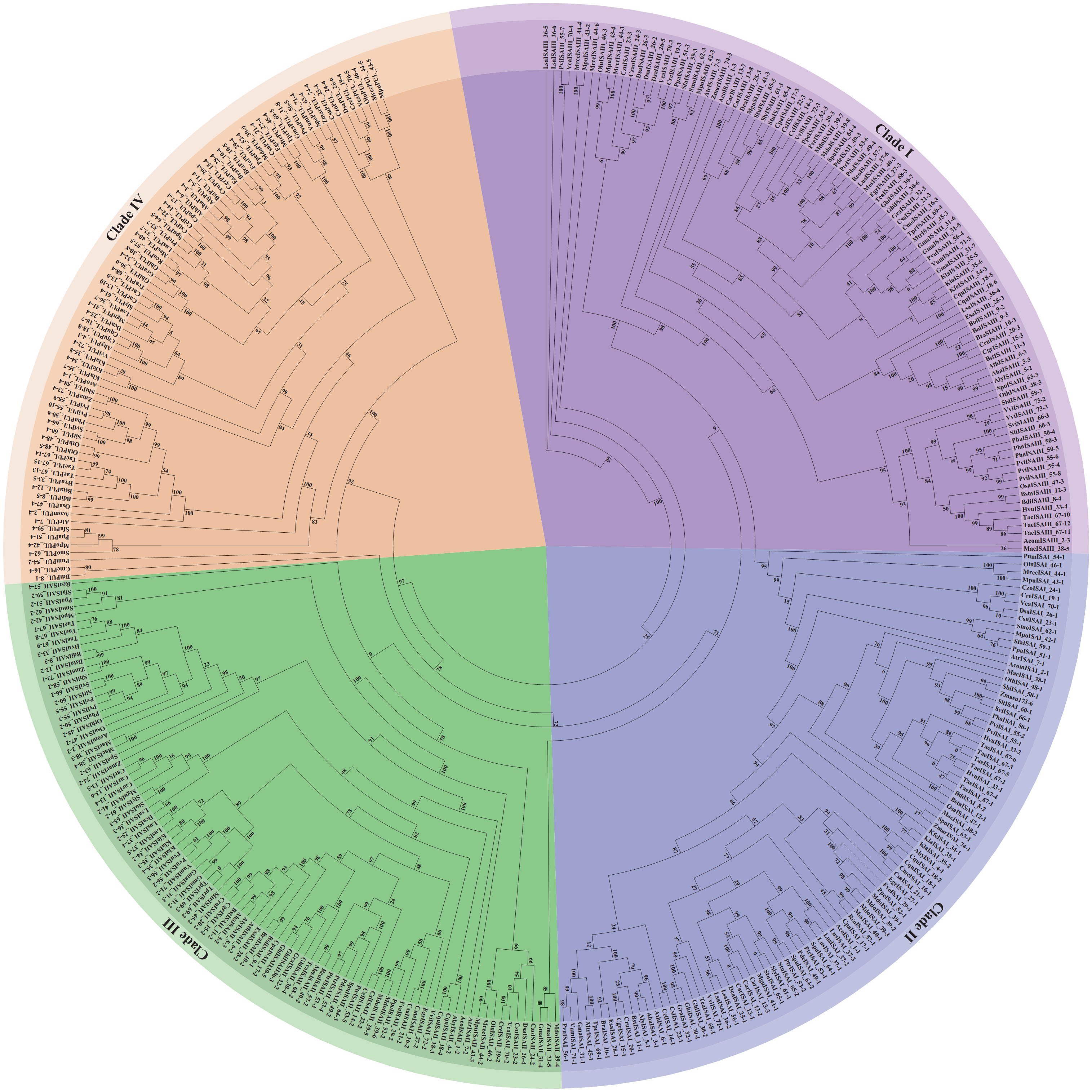
**Supplementary Fig. 13 Active site alignment of maize SBE isoforms, rice BEI and *Escherichia coli* glycogen branching enzyme.** Amino acid residues shown in different colours represent different active sites.


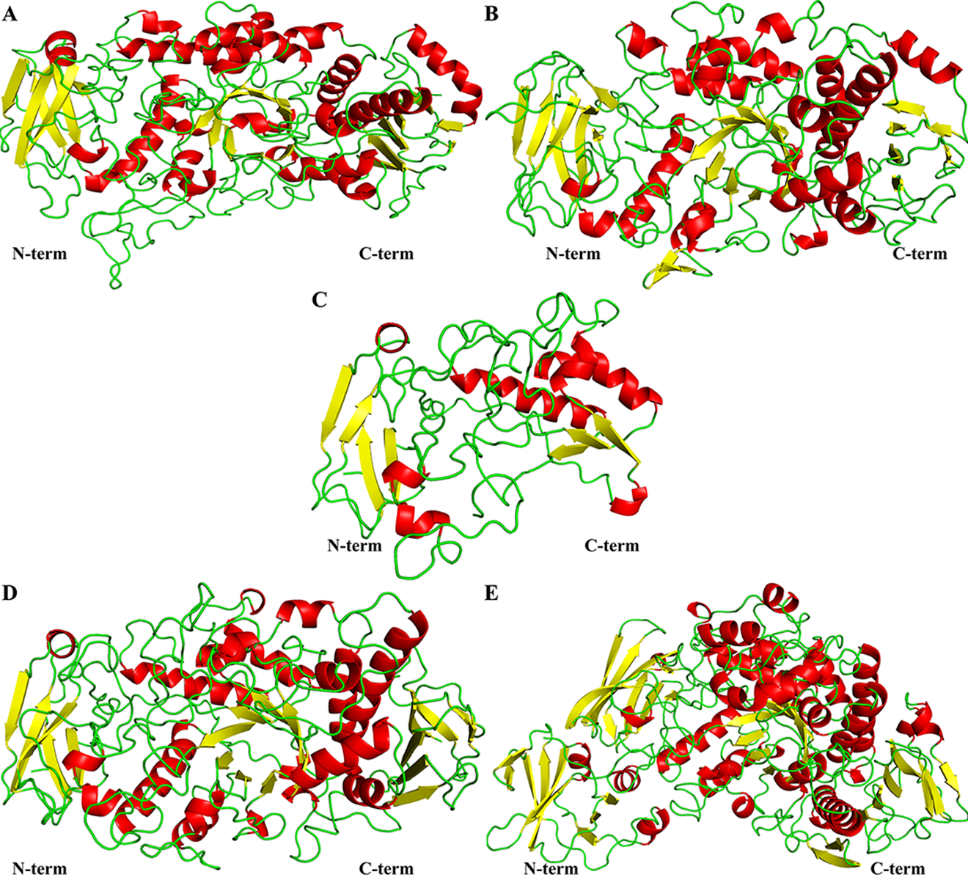
**Supplementary Fig. 14 Phylogenetic tree of DBEs.** Protein sequences of DBE in 74 plant species were divided into four clades. Clade I, clade II, clade III and clade IV represent ISAIII, ISAI, ISAII and PUL, respectively.


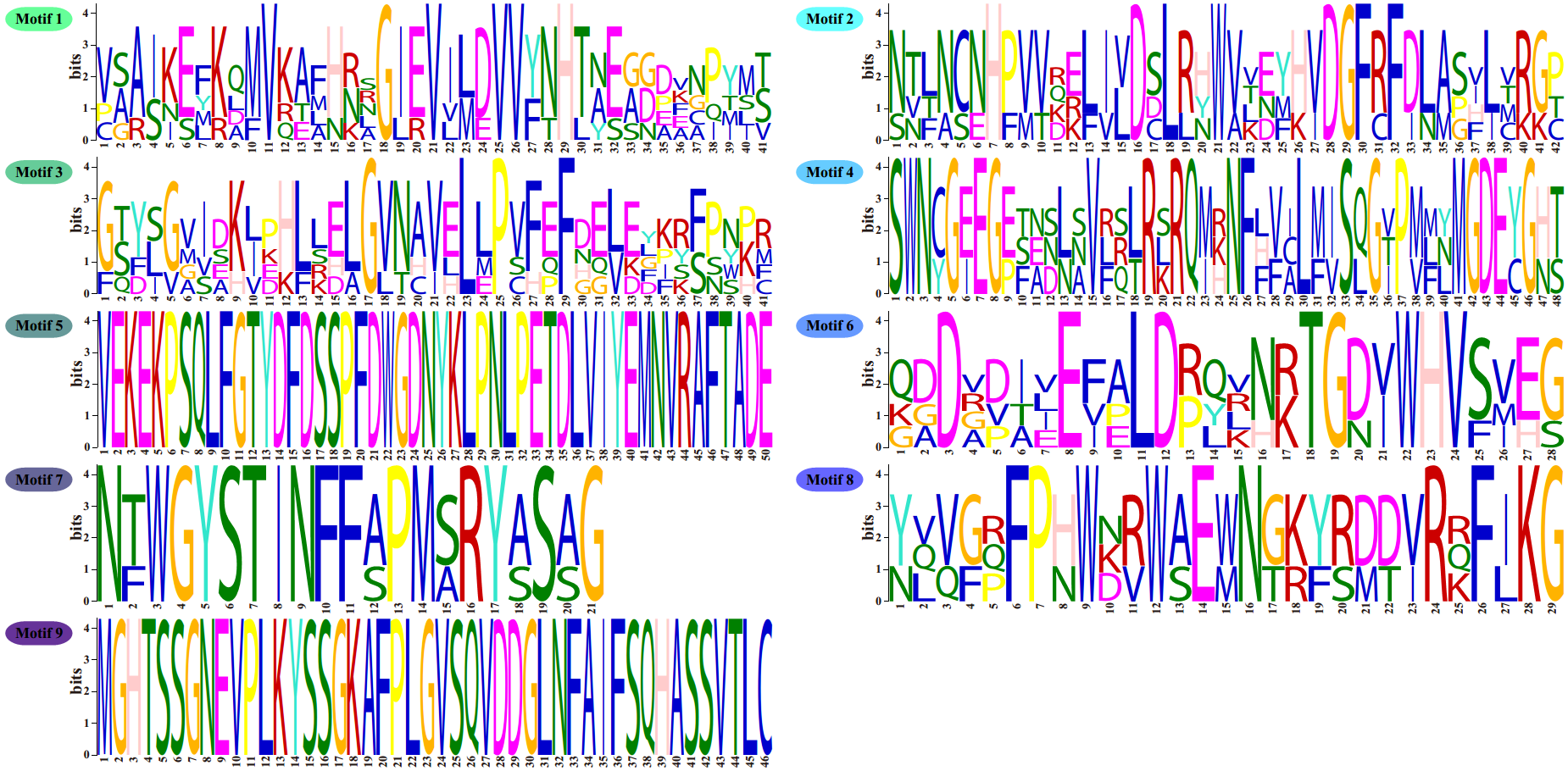
**Supplementary Fig. 15 Cartoon representing the secondary structural organizations of DBE isoforms.** Α-E represent the overall structure of su1, ISAII, ISAIIIb, ISAIIIa and PUL, respectively. α-helixes are coloured red, and β-folds are coloured yellow.


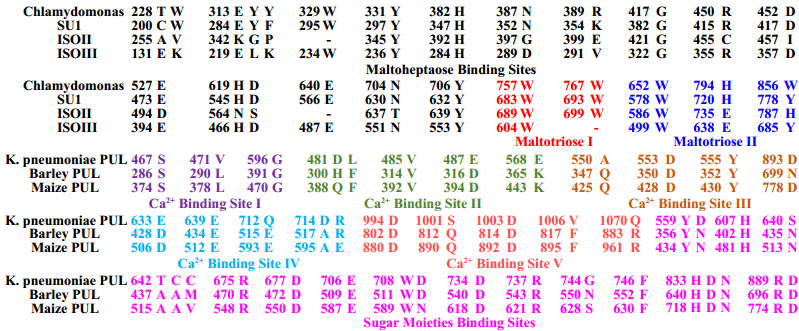
**Supplementary Fig. 16 Motif logo of DBE isoforms.**


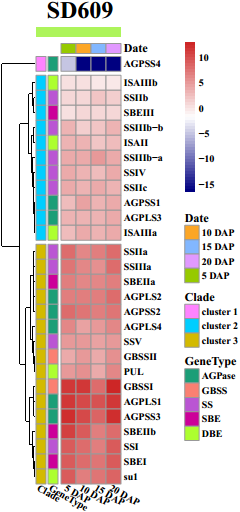
**Supplementary Fig. 17 Active site alignment of maize DBE isoforms, *Chlamydomonas* ISA1, PUL of *K. pneumoniae* and Barley.** Amino acid residues shown as different colours represent different active sites that interact with maize DBE isoforms.

**Supplementary Fig. 18 Expression patterns of starch synthesis**-**related genes in endosperm.** Gene expression data are shown for four development stages of SD609 endosperm. The scale bar shows the normalized FPKM values.
